# A landscape of recent advances in lipid nanoparticles and their translational potential for the treatment of solid tumors

**DOI:** 10.1002/btm2.10601

**Published:** 2023-11-09

**Authors:** Radu A. Paun, Sarah Jurchuk, Maryam Tabrizian

**Affiliations:** ^1^ Department of Biomedical Engineering, Faculty of Medicine and Health Sciences McGill University Montreal Quebec Canada; ^2^ Faculty of Dentistry and Oral Health Sciences McGill University Montreal Quebec Canada

**Keywords:** chemotherapy, drug combination, gene therapy, immunotherapy, lipid nanoparticles, solid tumors, translational limitations

## Abstract

Lipid nanoparticles (LNPs) are biocompatible drug delivery systems that have found numerous applications in medicine. Their versatile nature enables the encapsulation and targeting of various types of medically relevant molecular cargo, including oligonucleotides, proteins, and small molecules for the treatment of diseases, such as cancer. Cancers that form solid tumors are particularly relevant for LNP‐based therapeutics due to the enhanced permeation and retention effect that allows nanoparticles to accumulate within the tumor tissue. Additionally, LNPs can be formulated for both locoregional and systemic delivery depending on the tumor type and stage. To date, LNPs have been used extensively in the clinic to reduce systemic toxicity and improve outcomes in cancer patients by encapsulating chemotherapeutic drugs. Next‐generation lipid nanoparticles are currently being developed to expand their use in gene therapy and immunotherapy, as well as to enable the co‐encapsulation of multiple drugs in a single system. Other developments include the design of targeted LNPs to specific cells and tissues, and triggerable release systems to control cargo delivery at the tumor site. This review paper highlights recent developments in LNP drug delivery formulations and focuses on the treatment of solid tumors, while also discussing some of their current translational limitations and potential opportunities in the field.

**Translational Impact Statement**

Lipid nanoparticles have been successfully used in medicine as drug delivery vehicles leading to improved safety in patients. However, recent trends in oncology show that cancer treatments are multifactorial, sometimes requiring multiple drug combinations. Herein, we summarize recent advances in the use of lipid nanoparticles as multipurpose delivery vectors carrying at least one drug for the treatment of solid tumors. We further elaborate on the need to develop precision targeting of lipid nanoparticles to overcome some of the current limitations in the field.

## 1 INTRODUCTION

Cancer remains one of the leading causes of death to this date and can be broadly classified into hematological or solid malignancies.^1^ For both of these classes, patient survival rates have been steadily improving across most cancer types, with some exceptions attributed to more aggressive forms.^2^ While the incidence of some malignancies continues to decline due to preventive measures, such as reducing alcohol consumption and smoking, other cancers like invasive melanoma are becoming more common during clinical screenings in North America.^1^, ^3^ Similarly, significant therapeutic and diagnostic advances have led to a marked decline in the mortality rate for cancers like leukemia, while others, such as pancreatic cancer, continue to be fatal for the majority of patients.^1^, ^4^ In contrast to chemotherapy, which aims to kill rapidly dividing cells, the advent of personalized and precision medicine has enabled the development of (1) targeted therapeutics, which are small molecules that bind to and inhibit cancer‐specific proteins needed for survival and growth, (2) gene therapies, which typically deliver or silence genes of interest, and (3) immunotherapies, which aim to stimulate the immune system to attack and eliminate cancer cells. Exciting new developments in targeted therapies, using oncoprotein inhibitors such as asciminib and vemurafenib, gene therapies such as nadofaragene firadenovec and talimogene laherparepvec against bladder cancer and melanoma, respectively, as well as immunotherapies like chimeric antigen receptor‐engineered T and natural killer (CAR‐T/NK) cells, bispecific antibodies (Ab) targeting receptors expressed on cancer cells like CD20 or CD3 (odronextamab), and CTLA‐4 or PD‐1 immune checkpoint inhibitors (ipilimumab, nivolumab) have shown impressive preclinical and clinical results.^5^, ^6^, ^7^ However, some established solid tumors are more difficult to treat in part due to the complexity of the tumor microenvironment (TME), which contains several barriers that significantly limit the therapeutic efficacy of drug and cell therapies.

**FIGURE 1:**
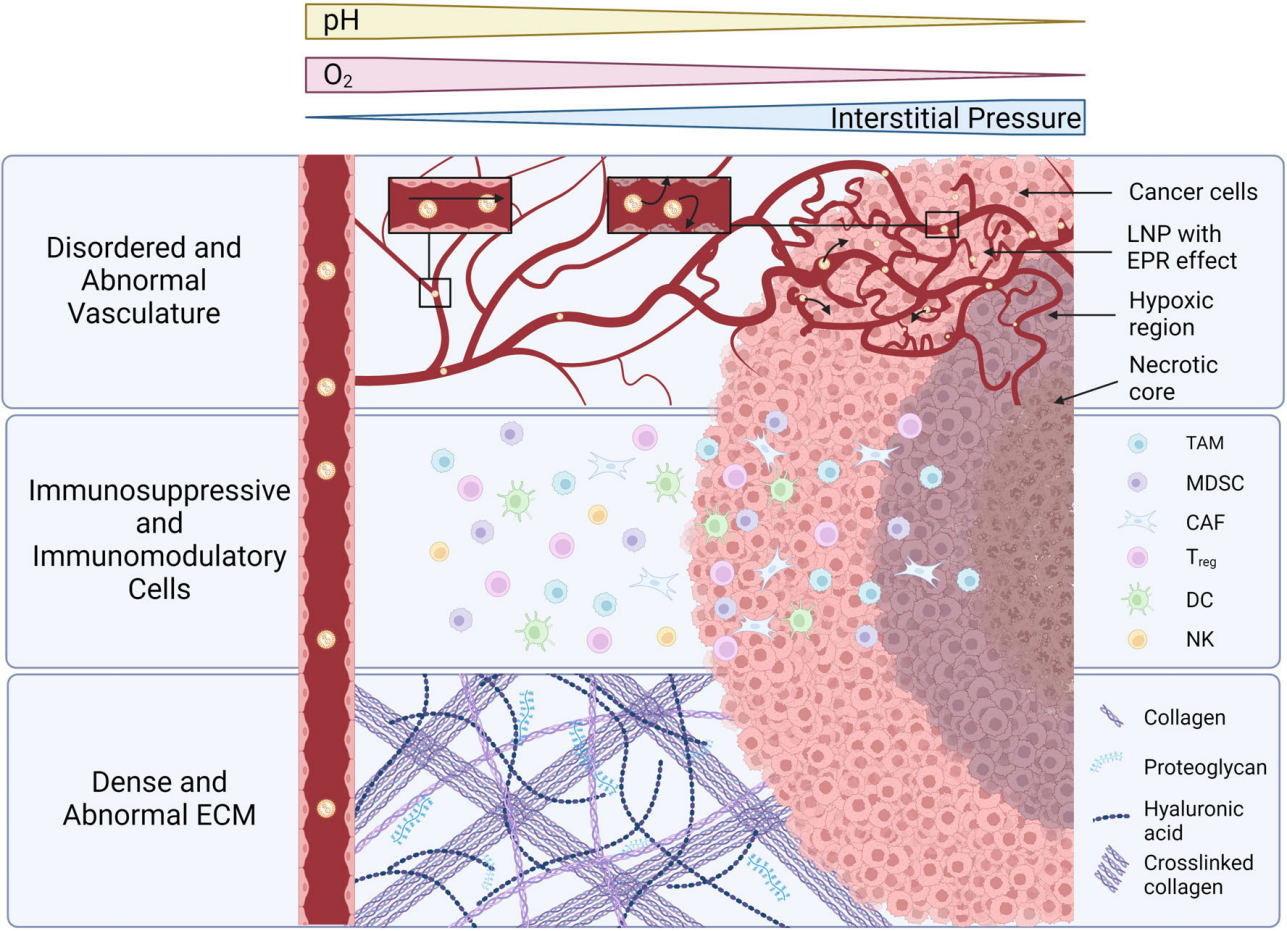
Schematic diagram illustrating the complexity of the tumor microenvironment and the various components that may contribute to nanoparticle sequestration. These include the enhanced permeation and retention effect (EPR) and the presence of scavenger cells such as tumor‐associated macrophages (TAM), as well as physical constraints such as a dense extracellular matrix (ECM). Created with BioRender.com.

The TME is a complex collection of cells and molecular products that have profound implications in disease progression, treatment resistance, and metastasis (Figure 1). Typically, the TME contains multiple immune and stromal cells, such as tumor‐associated macrophages (TAM) and cancer‐associated fibroblasts (CAF), which have been significantly reprogrammed by mutated cancer cells to create a pro‐cancer environment.^8^ While the TME is highly heterogeneous, depending on the cancer type and anatomical location of the tumor, common TME features include an acidic microenvironment due to an overactive metabolism, the presence of chronic inflammation leading to the recruitment of immunosuppressive cells, such as myeloid‐derived suppressor cells (MDSC) or regulatory T cells (T_reg_), as well as the upregulation of immune checkpoint receptors (e.g., PD‐L1, CTLA‐4) involved in the process of immunoediting. Immunoediting involves dynamic interactions between cancer and immune cells that result in the elimination of cancer cells, or an equilibrium state that can give rise to escape variants.^8^, ^9^ As a dynamic process, immunoediting can exert selective pressures inside the TME to drive the clonal selection of cancer cells with mutations that allow antigen escape or resistance to immunotherapy, such as mutations in the IFN/JAK/STAT pathway in refractory melanoma.^10^, ^11^, ^12^ Additionally, in some cancers, the excessive deposition of extracellular matrix, secretion of proteases and immunosuppressive enzymes may limit the penetration and activity of drugs or infiltrating lymphocytes thereby reducing their therapeutic efficacy.^13^, ^14^, ^15^ Furthermore, the aberrant vascular and lymphatic systems in the TME underline yet another aspect that may contribute to therapeutic resistance. As physiological blood circulation, and therefore tissue oxygenation, is reduced, it leads to pronounced hypoxia in poorly vascularized but highly proliferative tumor regions. Some of these alterations may result in the development of a metastatic niche, which promotes the colonization of cancer cells in other tissues, such as the lymph nodes. This has been recently shown to induce tumor immune tolerance and facilitate the dissemination of cancer cells to more distant sites for colonization.^16^ In some cases, tumors also have an elevated interstitial pressure as a result of dysfunctional lymphatic drainage, further exacerbating the limited diffusion rate of drugs and reduced infiltration of anticancer immune cells.^17^ These therapeutic limitations of the TME are compounded by potentially serious adverse events due to on‐ and off‐target toxicities of therapeutics, limiting their dosing and combinatorial potential in the clinic.^18^, ^19^, ^20^, ^21^

The chaotic nature of the tumor vasculature, as well as other characteristics of the TME, such as high acidity, hypoxia, and immunosuppression, are features that can be exploited by next‐generation drug delivery systems to improve the therapeutic action of anti‐neoplastic therapies. More specifically, nanotechnology has an important role to play in this context as it can address some of the limitations set by the TME with respect to freely soluble drugs. The Enhanced Permeation and Retention (EPR) effect is a well‐documented phenomenon that is due to irregular angiogenesis occurring within solid tumors, whereby larger macromolecules and nanoparticles are preferentially retained in the tumor tissue.^22^, ^23^ Historically, the EPR effect has been attributed to large fenestrae and discontinuities in the tumor endothelium leading to the passive accumulation of nanoparticles in the tissue.^24^ While bulk diffusion through fenestrae would be limited for both free drugs and nanoparticles, recent evidence suggests that the delivery of nanoparticles into tumors is more complex, with nanoparticles appearing to be predominantly delivered into the tumor tissue by specific endothelial cells termed nanoparticle transport endothelial cells (N‐TEC) using an active transport mechanism.^25^, ^26^ These findings are consistent with the known heterogeneous distribution of nanoparticles within tumors after systemic administration, as well as the different therapeutic effects observed when drugs are administered freely in solution compared with a nanoparticle formulation, where the drugs seem to kill a larger repertoire of TME cells when delivered using nanoparticles.^27^ Preliminary evidence for this observation could be attributed to the limited diffusion of freely circulating drugs once they cross the endothelial barrier, whereas nanoparticles can be sequestered by TAMs and redistributed within the tumor where they can be potentially released at a later time.^28^, ^29^ This process, if validated, could address some of the diffusivity issues at the tumor‐endothelial interface, as TAMs may infiltrate deeper into the tumor after nanoparticle sequestration and act as a slow‐release drug depot, thus underlining the importance of considering non‐cancerous cells in the TME when designing nano‐based therapies. Additionally, one can envision the development of nanoformulations that are chemically programmed to respond to physicochemical changes in the TME, such as pH or oxygen tension, to preferentially release therapeutics once in the TME, with the overall goal of reducing systemic toxicity.^30^ As noted, there are multiple advantages to using nanoparticles for cancer therapy, especially when it comes to lipid‐based formulations as they are one of the most successful drug delivery systems developed to date.

**FIGURE 2:**
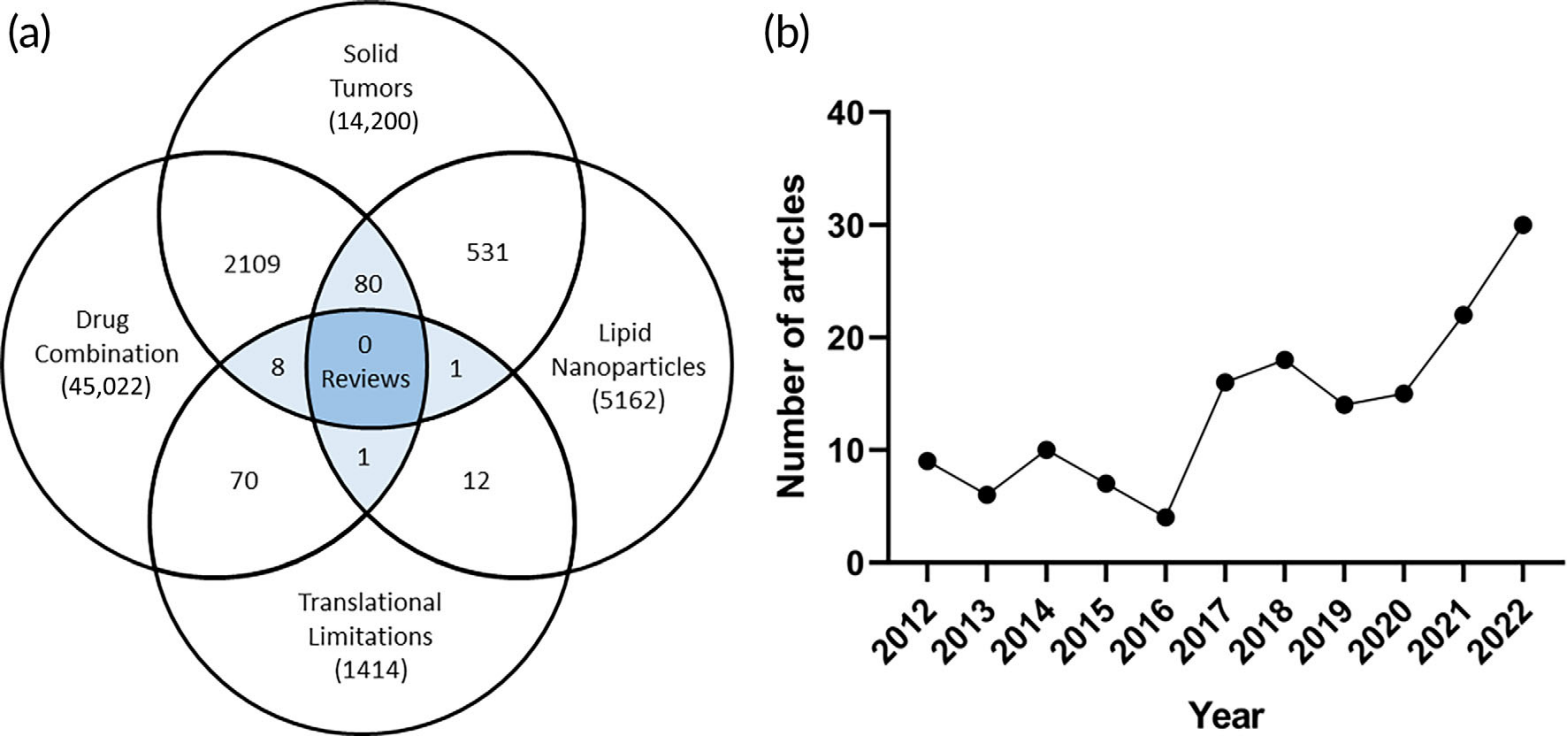
(a) Venn diagram showing the number of reviews found by performing a SCOPUS search using the keywords “solid tumors,” “lipid nanoparticles,” “translational limitations,” and “drug combination,” suggesting that currently, there are no reviews in the literature that discuss these points together. (b) Graphical representation of the primary research papers included in this review by year.

Due to the intricacies of the TME, and its dynamic nature, developing nanotherapeutics is becoming increasingly more complex. Scientists and engineers must balance multiple design constraints that result in stable nanocarriers with various therapeutic approaches potentially involving the combination of more than one active pharmaceutical ingredient (API) in the same system. In addition, the more complex the nanoformulation, the more difficult the safety and efficacy evaluation by government health agencies such as the Food and Drug Administration (FDA). Regulatory requirements can significantly limit the translational potential of complex nanotherapeutics, and given the large investment required for preclinical and clinical testing, they should be considered from the early stages of development to improve the bench‐to‐bedside pipeline and reduce controllable risk as much as possible. In this review, we focus on contextualizing the most recent developments in lipid nanoparticle drug delivery technology involving at least one API for the treatment of solid tumors with their intrinsic translational limitations since there are currently no reviews that consider all of these aspects (Figure 2a). We searched for papers published between 2012 and 2022 for relevant advances in lipid nanoparticle‐based drug delivery systems for the treatment of solid cancers and included nearly 200 original contributions in this review paper (Figure 2b). The first section of the review briefly defines and summarizes the major types of lipid nanoparticles (LNP) that have been developed and studied to date. Additionally, the motivation for writing such a review was to place these nanoparticles in the context of their routes of administration and the delivery of multiple types of therapeutic cargo. Thus, a considerable portion of this review discusses the fate of multi‐dimensional LNPs specifically designed for solid tumor therapy, and their relevant delivery routes, enabling them to cross multiple pathophysiological barriers associated with the complexity of the TME. The review starts by discussing advances in the design of various types of LNPs used for locoregional delivery, followed by presenting the systemic delivery of LNPs without (passive delivery) or with targeting (targeted delivery) ligands with the aim of significantly reducing off‐target effects, which is an important consideration for the extensive use of more complex systems, such as gene‐based therapeutics. Finally, the review recapitulates some of the current limitations in the field with respect to the translational aspect of LNPs and outlines future opportunities for the development of more effective delivery systems for the treatment of solid tumors.

## 2 LIPID NANOPARTICLES AS DRUG DELIVERY VEHICLES

**FIGURE 3:**
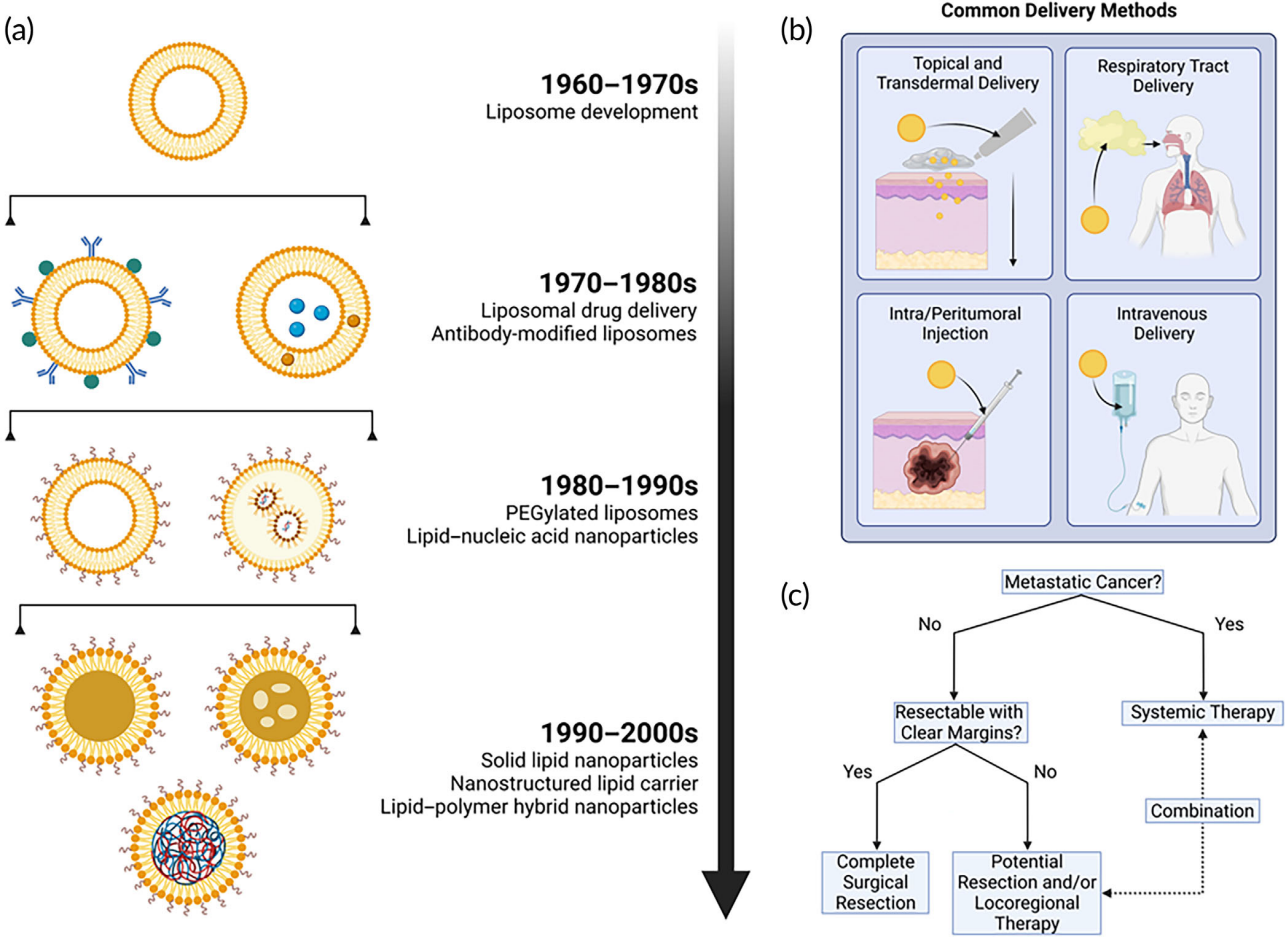
(a) Major types of lipid nanoparticles and their development timeline. (b) Various physical routes that can be used for the delivery of lipid nanoparticle formulations to patients. (c) Decision tree outlining the process of selecting a drug delivery route for the treatment of solid tumors. Created with BioRender.com.

Due to their biocompatible and versatile nature, LNP vectors represent an attractive approach to drug delivery in oncology. As their name implies, LNPs are nanoparticles constructed using phospholipids, or phospholipid derivatives, which endow them with unique physicochemical properties. They currently represent some of the most successful nano‐based therapeutic and vaccination vehicles approved by regulatory agencies for use in clinical settings.^31^ There are multiple types of LNPs as shown in Figure 3a, each with their own advantages and limitations. LNPs are formulated with the aim of increasing their stability in plasma. This typically involves the addition of polyethylene glycol (PEG) chains to composite phospholipids, a practice known as PEGylation,^32^ which prolongs the circulation half‐life of LNPs by sheltering them from protein aggregates, opsonization, and premature clearance.^33^ However, some recent evidence has shown that PEGylation may have some limitations due to a potential immune response to the PEG chains resulting in the secretion of anti‐PEG antibodies. This could lead to an accelerated blood clearance of PEGylated nanoparticles when administered more frequently.^34^, ^35^ Nonetheless, PEGylation may have other beneficial effects on the TME, as shown by Wouters et al., who investigated the effects of various nanoparticle carriers with respect to the TME of ovarian cancer.^36^ The authors found that PEGylated LNPs have a favorable immune profile and do not induce local innate immune suppression like other carriers, such as polylactic‐*co*‐glycolic acid (PLGA), calcium carbonate microparticles, hydroxyapatite, or polystyrene. While questions still remain about the detection techniques of anti‐PEG antibodies and the way that PEG molecules interact with antibodies when bound to nanoparticles, there are strategies to potentially limit the accelerated clearance of PEGylated nanoparticles by: (1) overwhelming the clearance system of the body before, or concurrent with, the therapeutic injection, (2) shedding PEGylated lipids after in vivo administration, or (3) completely replacing PEG with other materials such as zwitterionic polymers.^37^, ^38^, ^39^, ^40^, ^41^ The choice of formulation is highly dependent on the delivery route (Figure 3b), which is itself dependent on the type and stage of the treated cancer. Typically, metastatic cancers are treated with drugs delivered systemically to ensure proper perfusion of all tissues that may harbor metastatic niches, while more localized disease can be treated with surgery alone, or a combination of various methods depending on the tumor characteristics (Figure 3c). Some of the most common types of LNP formulations are discussed in more detail in the following sections.

### 2.1 Liposomes

Liposomes are small synthetic vesicles larger than 20 nm and containing at least one phospholipid bilayer typically surrounding an aqueous core. Liposomes have been extensively reviewed in the literature as they are one of the first developed nano‐based drug delivery systems.^42^, ^43^, ^44^ Advances in liposomal manufacturing and encapsulation methods have resulted in multiple commercial formulations containing common cytostatic drugs, such as doxorubicin (Doxil®, Caelyx®, Lipodox®), vincristine (Marqibo®), irinotecan (Onivyde®), and cisplatin (Lipoplatin™), which have overall improved toxicity profiles compared with their unencapsulated counterparts.^31^, ^45^, ^46^ The ability of liposomes to encapsulate both hydrophilic (in the core) and hydrophobic (in the core as precipitates or the bilayer) molecules within a single particle makes them versatile for synergistic drug combinations, which is the case with Vyxeos®, a clinically approved liposomal combination of daunorubicin and cytarabine for the treatment of leukemia.^47^

### 2.2 Solid lipid nanoparticles and nanostructured lipid carriers

Solid lipid nanoparticles (SLNs) consist of a solid lipid core, mainly containing glycerides, fatty acids, sterols, or waxy molecules that are surface stabilized by emulsifiers.^48^, ^49^ SLNs can solubilize and retain lipophilic molecules into their core, allowing for drug delivery of compounds that would be poorly stabilized by other vectors, such as liposomes or oil‐in‐water nanoemulsions. On the other hand, nanostructured lipid carriers (NLC) contain a lipidic–liquid interface, usually consisting of oils like oleic acid, and typically embedded within a solid core such as an SLN.^50^, ^51^ NLCs can further enhance the solubility and stability of certain compounds that are not well solubilized in the solid core of SLNs, making them a suitable delivery vector for the potential co‐encapsulation of drugs in either the oil phase, the solid phase, or both depending on the matrix mixture and drug properties.

### 2.3 Lipid‐polymer hybrid nanoparticles

Lipid‐polymer hybrid nanoparticles (LPHN) are a newer form of LNPs that typically contain a polymeric component as the core of the particle surrounded by a lipid monolayer that acts as a surfactant, but other configurations are also possible.^52^, ^53^ The lipid monolayer serves as a stabilization unit that can prevent the encapsulated drugs from freely diffusing out of the polymeric system, while at the same time adding extra functionalities to the surface by allowing further chemical modifications.^52^ There are many advantages to using polymer‐based nanoparticles for drug loading and release. First, polymers are highly modular and can be designed as linear or branched; inert or biodegradable; hydrophilic, hydrophobic, or amphiphilic; thus, adding extra layers of complexity to the system, especially when more than one polymer is used simultaneously.^54^, ^55^, ^56^, ^57^ For encapsulation within LPHNs, drugs can be grafted onto the polymer chains or loaded within the polymer matrix where the polymer behaves as an excipient to stabilize the molecular interactions of various small molecules, nucleic acids, or proteins, with the goal of improving co‐encapsulation stability and yield.^58^ In addition, polymers can be constructed as smart materials that can respond to changes in the environment (e.g., pH, oxygen concentration) or to exogenous triggers (e.g., mechanical, photo‐stimulation).^30^

### 2.4 Lipid‐nucleic acid nanoparticle complexes

Lipid‐nucleic acid nanoparticles (LNAN) are lipid‐based nanoformulations of various types of nucleic acids, such as plasmids, mRNA, siRNA, and so on, with the scope of protecting them from degradation and improving their intracellular delivery.^59^, ^60^, ^61^, ^62^ Since both DNA and RNA are negatively charged, ionizable lipids, or other positively charged stabilizers, such as polymers or dendrimers, have been designed to form complexes with nucleic acids, which generally form the core of the LNAN, in addition to the standard components (cholesterol, phospholipids and PEG‐lipids).^59^, ^63^, ^64^ The ionizable lipids play another role, which is to facilitate the endosomal escape of the nucleic acids once internalized by the cell through endocytosis. However, the mechanism of endosomal escape is not yet entirely elucidated, but some hypotheses are gaining traction.^65^ LNANs have recently gained mainstream attention during the COVID‐19 pandemic as mRNA delivery vehicles (Cominarty®, Spikevax™) for the prophylactic vaccination against the SARS‐CoV‐2 virus, demonstrating a remarkable safety and efficacy profile.^66^, ^67^ These types of nucleic acid delivery systems can also be applied to several other disease areas, including for the treatment of cancer.^68^, ^69^

### 2.5 Other formulations

There are several other types of LNPs that can be constructed, such as niosomes using non‐ionic surfactants,^70^ ethosomes formulated with ethanol,^71^ flavosomes containing flavonoids,^72^ cubosomes as nanoparticles structured in a bicontinuous cubic liquid crystalline phase,^73^ tocosomes containing vitamin E derivatives,^74^ and others.^75^, ^76^, ^77^, ^78^, ^79^, ^80^, ^81^ For example, Marwah et al. developed a formulation to deliver deformable liposomes, also known as elastic liposomes, to dermal cells.^82^ Deformable liposomes contain a less rigid membrane due to the presence of surfactants in the lipid bilayer, which act as edge activators.^83^ Compared with regular liposomes, deformable liposomes are typically formulated to enhance drug delivery into or across different skin layers, which is achievable due to their stretchable and malleable nature.^84^, ^85^

## 3 FORMULATIONS FOR THE LOCOREGIONAL DELIVERY OF LIPID NANOPARTICLES

Locoregional delivery of nanoparticles is not subject to the barriers of more common delivery routes, such as in oral drug delivery with the first‐pass metabolism of nanocarriers by the liver, or issues with the gastric stability of nanoparticles and their intestinal permeation into the systemic circulation. This underlines the complexity of designing oral lipid‐based nanoparticle formulations for local or systemic delivery, which are discussed elsewhere.^113^ With local LNP‐based drug delivery, there is direct exposure of tumors to selected drugs at higher concentrations than would normally be found with systemic administration, which may have significant advantages due to reduced systemic risk.

### 3.1 Topical and transdermal delivery

#### 3.1.1 Topical delivery

Topical drug delivery systems can act on different layers of the skin, from the superficial epidermis to the subdermal layer, and can also work transdermally to deliver drugs into the systemic circulation.^114^ Due to the delivery location, the most common solid tumors treated by topical delivery systems are primary skin cancers or secondary skin metastases. Patient compliance is typically high with this delivery method; however, both dermal and transdermal delivery routes, as well as many locoregional formulations, exhibit low drug penetration rates, therefore continuous efforts are being made to improve the delivery performance (Table 1).^115^ Drugs must first pass through the stratum corneum, typically the most impervious layer of the skin, and then across the epidermal and dermal layers to reach their intended targets, making the design of such systems more challenging.^116^ Drug delivery is typically achieved through various forms of gels, creams, ointments, or patches in which the active ingredients are incorporated. For example, a nanocream formulation containing dacarbazine‐loaded nanoparticles was developed for the topical treatment of superficial melanoma, and since dacarbazine is poorly soluble in water, it was encapsulated into stearic acid‐based LNPs.^92^ Topical formulations containing LNPs have also been evaluated in clinical trials, including a phase I/II clinical trial (NCT03101358) that studied the safety and efficacy of a liposomal paclitaxel‐based ointment. The study showed that a dose of up to 2.0% of liposome‐encapsulated paclitaxel in an anhydrous ointment was safe and well tolerated.^117^ The subjects all had cutaneous malignancies, primarily of breast cancer origin, which stabilized or improved over the 28‐ or 56‐day study period. Furthermore, there was negligible systemic paclitaxel absorption, which shows good locoregional control of toxicity.

**TABLE 1:** Summary of locoregional methods and formulations for LNP‐based drug delivery to solid tumors.

Delivery	Cancer	Cell	Model	LNP type	Mechanism of action	Drugs	Date/Ref
Topical or transdermal	Breast	BT474	Ectopic	PEG‐PEI Lipoplex	Estrogen receptor modulation	Tamoxifen	2016^86^
		MCF‐7	In Vitro	Transferosome	Estrogen receptor modulation	Raloxifene	2021^87^, ^88^
				Elastic Liposome	Immunomodulation	Luteolin	2021^89^
	Colorectal	CT‐26	Ectopic	PEG‐PEI‐Lipoplex	Immunomodulation, estrogen receptor modulation	Curcumin, Tamoxifen	2012^90^
	Melanoma	A375	In Vitro	Pheroid™	Thymidylate synthase antagonism	5‐FU	2015^91^
		B16F1		Nanoemulsion	DNA alkylation	Dacarbazine	2017^92^
		B16F10	Ectopic	Cationic Lipoplex	Immunomodulation, inhibition of STAT3 expression	Curcumin, STAT3 siRNA	2018^93^
				PEG‐PEI‐Lipoplex	DNA intercalation, TLR 9 agonism	DOX, CpG	2023^94^
Inhalable	Breast	4T1	Metastatic	Liposome	STING‐mediated IFN response	cGAMP	2019^95^
	Lung	A549	Orthotopic	Liposome	XPB antagonism, CA IX targeting	Triptolide, αCA IX	2018^96^, ^97^
		M109		SLN	Microtubule stabilization	Paclitaxel	2018^98^
	Melanoma	B16‐OVA	Metastatic	Liposome	STING‐mediated IFN response	cGAMP	2019^95^
		B16F10		Liposome	TLR 9 agonism	CpG	2021^99^
Intratumoral or peritumoral	Breast	4T1	Orthotopic	Liposome	TLR 7 agonism	Imiquimod	2019^100^
			Ectopic + Metastatic	iGel	Interfering with DNA synthesis, TLR 7 agonism, macrophage depletion, PD‐1 blockade	Gemcitabine, Imiquimod, Clodronate, αPD‐1	2019^101^
	Cervical	TC1	Ectopic + Metastatic				
	Colon	CT26	Ectopic	Liposome	TLR 3 agonism, photosensitization	Poly I:C, ICG	2019^102^
		CT26, MC38	Ectopic	Liposome	Photosensitization, PD‐1 and TIM‐3 blockade	ICG, αPD‐1, αTIM‐3	2020^103^
		CT26		Cationic Liposome	Immunogenic cell death	Plasmid DNA	2020^104^
	Fibrosarcoma	H‐1080	Ectopic	Liposome	DNA intercalation, death receptor 4/5 agonism	DOX, TRAIL	2019^105^
	Gastric	BGC‐823	Ectopic	Liposome	TLR 3 agonism	Poly I:C	2013^106^
	Melanoma	B16	Ectopic + Metastatic	Liposomal gel	TGF‐β receptor antagonism, immunomodulation	LY364947, IL‐12	2012^107^
			Ectopic	Liposome	Microtubule stabilization, immunomodulation	Paclitaxel, IL‐12	2013^108^
				Liposome	Microtubule stabilization, photosensitization	Poly I:C, ICG	2019^102^
				Liposome	Photothermal release of DOX (DNA intercalation), immunomodulation	Nanogold DOX, vaccine‐based nanoparticles	2021^109^
				Liposome	Photothermal therapy with DOX release (DNA intercalation), antigen presentation via liposomes, PD‐L1 blockade	Nanogold DOX, antigen‐capturing liposomes, αPD‐L1	2021^110^
				LNAN	Secretion of proinflammatory IL‐12 and IL‐27	IL‐12 and IL‐27 mRNA	2022^111^
	Ovarian	ID8	Ectopic	Liposome	TLR 7/8 agonism, PD‐1 blockade	Resiquimod, αPD‐1	2021^112^
				Liposome	DNA intercalation	DOX	2022^36^

**FIGURE 4:**
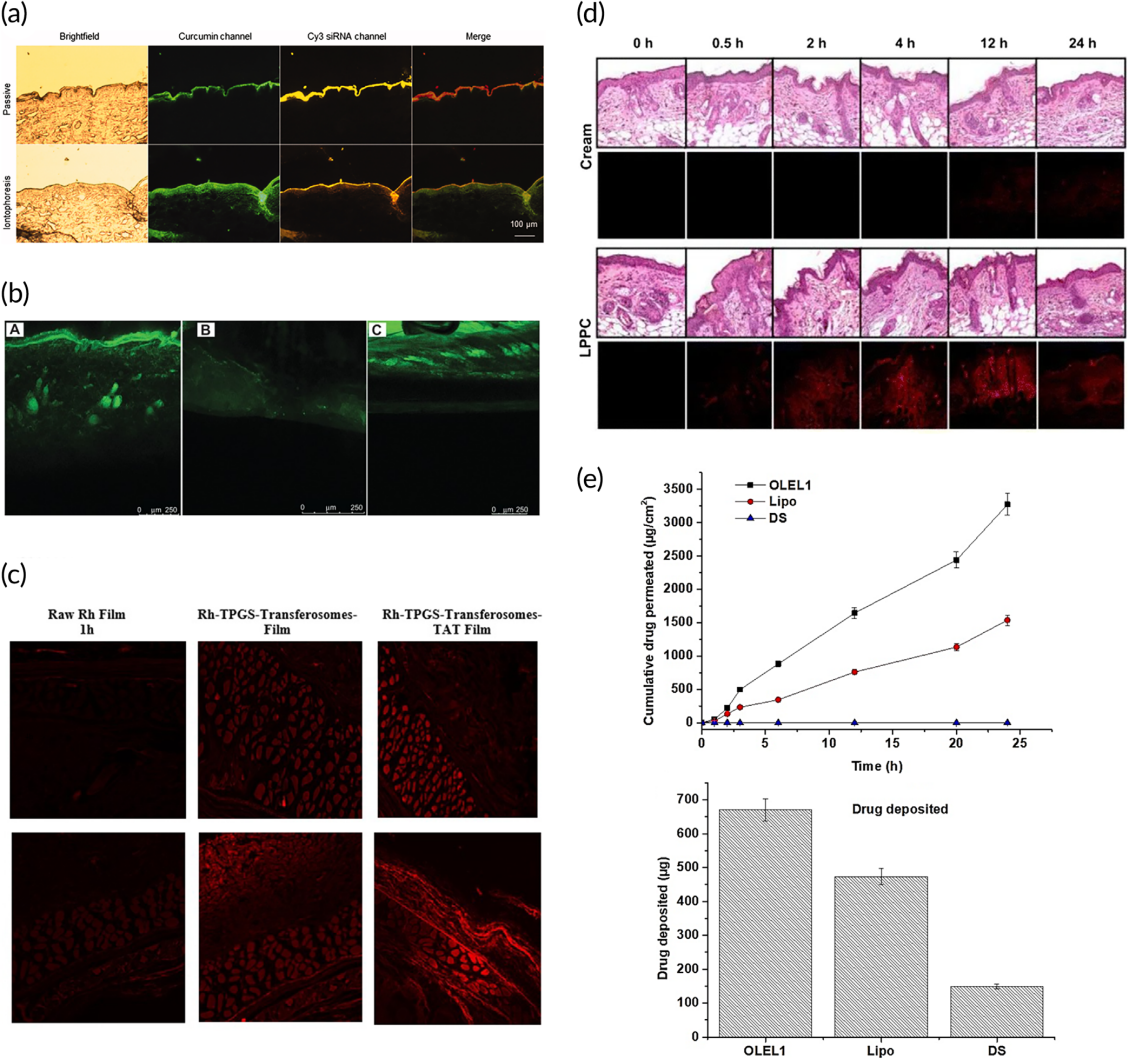
Topical and transdermal delivery of lipid nanoparticles. (a) Micrographs of skin cryosections after treatment with the curcumin‐loaded liposome–siRNA complex in the presence or absence of iontophoresis. Scale bar = 100 μm.^122^ (b) Confocal images after application of 6‐coumarin‐loaded LNP system. RTV13, raloxifene transferosomes (a), liposomes with raloxifene (b), and ethanolic PBS with raloxifene (c).^124^ (c) Fluorescence images for the permeation of raw Rh transdermal film (left column), and Rh‐TPGS‐Transferosomes Film (middle column) and Rh‐TPGS‐Transferosomes‐TAT Film (right column) through rat skin after 1 and 4 h. Magnification ×400.^88^ (d) In vivo skin permeation of LPPC/DiI or cream/DiI (red fluorescence) after treatment in BT474 tumor‐bearing mice.^86^ (e) Ex vivo LUT release pattern of the optimized elastic liposome formulations (OLEL1) as compared with conventional liposomes (lipo) and drug solution (DS) over a period of 24 h; and drug deposition study of OLEL1, lipo, and DS into the skin after 24 h of permeation study.^89^ Figures are reprinted with publishers' permission.

In addition to the standard topical formulations, there are a variety of chemical enhancers that can optimize drug permeability, retention, and deposition into the skin, as nanoparticle approaches mostly aid with drug solubility and stability.^118^ Pheroid™ is a patented technology of a triple oil/water/gas phase combination containing suspensions of lipid‐based submicron and micron‐sized structures.^91^ In a study by Chinembiri et al., Pheroid™ was used to entrap and deliver 5‐fluorouracil (5‐FU) to human melanoma cancer cells (A375). The efficacy of 5‐FU in the Pheroid™ lotion to induce apoptosis of cancer cell was higher when compared with plain lotion or no treatment control. Penetration results also demonstrated significantly higher permeation using in vitro excised human skin for the 5‐FU Pheroid™ lotion, as well as better diffusion per unit area.^91^ Alternatively, active drug delivery methods, such as mechanical force or microneedle technology can be used to enhance the penetration depth of nanoparticles into the skin.^119^, ^120^, ^121^ Jose et al. evaluated the efficacy of a cationic liposomal complex in mouse models of melanoma.^93^, ^122^ Because of the limited permeation through the skin, they used iontophoresis, which involves the application of a voltage gradient resulting in the electrophoresis of charged particles across the skin. The authors developed a liposomal system with curcumin as an anticancer compound and an anti‐STAT3 siRNA to interfere with protein translation. Curcumin was encapsulated into 1,2‐dioleoyl‐3‐trimethylammonium propane (DOTAP) based cationic liposomes, which were then complexed with the anti‐STAT3 siRNA. On excised porcine skin models, liposomes with the iontophoresis application penetrated up to 160 μm into the skin, compared with 50 μm for those without iontophoresis (Figure 4a). In in vivo mouse models of melanoma, the iontophoretic administration of the lipoplex showed a significant reduction in tumor volume and tumor weight compared with the control group. The authors also compared the difference between the administration of the liposomal complexes via intratumoral injection and their administration by iontophoresis but found that there was no significant difference between the two in terms of outcomes. However, even physical methods have their limitations when it comes to diffusion barriers.^123^

#### 3.1.2 Transdermal delivery

Transdermal delivery is a technique used to deliver drugs to sties beyond the upper layers of the skin to improve compliance and reduce side effects, which can be facilitated by various types of formulations.^125^ While transdermal delivery does not achieve a delivery efficacy and bioavailability comparable to systemic delivery methods, some transdermal systems containing drugs with molecular weights <500 Da have been previously approved by the FDA.^126^ However, lipid nanoparticles can be developed to help small drugs better cross skin barriers by temporarily altering their physicochemical properties. An important transdermal LNP‐based formulation is the use of deformable or elastic liposomes, also known as transferosomes. Mahmood et al. designed a transdermal transferosome formulation to improve the bioavailability of raloxifene, which is commonly used to treat breast cancer and osteoporosis.^124^ The authors used ex vivo rat skin to compare formulations of raloxifene‐loaded transferosomes, conventional liposomes, and a vehicle solution and found that the transferosomes were significantly better at permeating and depositing the drug in the skin than any of the other options (Figure 4b). Similar studies compared the delivery of raloxifene to rat skin using a gel, patch, or transferosome formulation. Prolonged drug release over 48 h and a better permeation profile with no lag time were observed with transferosomes compared with the patch or gels.^127^ In addition, improved intracellular delivery of raloxifene transferosomes is reported when tocopheryl polyethylene glycol 1000 succinate (TPGS) was grafted as a surface‐active agent resulting in increased solubility and permeability of the formula.^88^ The authors used a cationic cell‐penetrating peptide from the HIV‐1 virus (TAT) to enhance the translocation of the drugs into the cells. Ex vivo permeation studies on rat skin showed that raloxifene‐loaded TPGS‐transferosomes loaded with TAT had a significantly higher percentage of diffused drug, and that TAT played a significant role in enhancing skin penetration (Figure 4c). In vitro cytotoxicity studies on breast cancer cells showed that the IC_50_ value of the raloxifene‐loaded TPGS‐transferosome‐TAT formulation was significantly lower than that of the raloxifene alone, corresponding to a 42% increase in cytotoxicity for the raloxifene‐TAT formulation compared with raloxifene alone.

Lin et al. showed that a tamoxifen‐encapsulated lipoplex can be used for transdermal delivery to treat breast cancer in a subcutaneous mouse model.^86^ Tamoxifen is a small molecule drug commonly used to treat estrogen receptor‐positive breast cancer by modulating estrogen receptor signaling. A cationic polyethylene glycol‐polyethyleneimine lipoplex (liposomal PEG‐PEI complex or LPPC), was used due to its apparent rapid tissue penetration capabilities.^90^, ^94^ In vivo studies showed that local delivery of the tamoxifen‐loaded LPPC increased drug accumulation in the subcutaneous tumor compared with a cream control as shown in Figure 4d. Further, the construct inhibited tumor growth up to 82% compared with controls with little to no cytotoxicity or irritation to the surrounding skin cells. In another study, Altamimi et al. loaded luteolin, an immunomodulatory compound, into elastic liposomes and investigated their effect on breast cancer compared with conventional liposomes or unencapsulated drug.^89^ The elastic liposome formulation showed the best cumulative drug permeation through ex vivo rat skin, and higher drug deposition than either conventional liposomes or drug alone, as well as a slower lag time (Figure 4e).

### 3.2 Respiratory track delivery

Respiratory track delivery of drug‐loaded LNPs is a direct method for the treatment of various pulmonary and respiratory diseases that relies on the inhalation of aerosolized drug formulations. With a large absorptive surface area, the lungs provide a rapid and widespread delivery pathway while reducing systemic clearance and metabolism.^128^ Local delivery to the lungs also minimizes the risk of systemic adverse events that can limit a drug's therapeutic index. Resultingly, the dose required for equivalent effects by systemic delivery is typically lower for LNPs delivered by inhaled lipid nanoparticles.^98^ Inhaled LNPs can be designed for higher tumor selectivity and optimized for sufficient residence time in the lungs. There are, nonetheless, several physiological challenges associated with pulmonary delivery. The heterogeneous structure of the respiratory tract can affect drug delivery, with various resident cell types exhibiting different nanoparticle uptake and sequestration rates.^129^ Further, clearance mechanisms of the lung that protect its mucosal layer must be considered, especially in diseased lungs. Pathological mucus is typically more dehydrated and viscous, which significantly alters its protein concentration and charge, which in turn can reduce the penetration of lipid nanoparticles.^129^ This can be overcome through various coatings and conjugations, an example of which is the coating of chitosan residues onto liposomes.^98^ In acidic formulations, chitosan is protonated, thereby becoming positively charged and more capable of passing through the negatively charged mucins.

**FIGURE 5:**
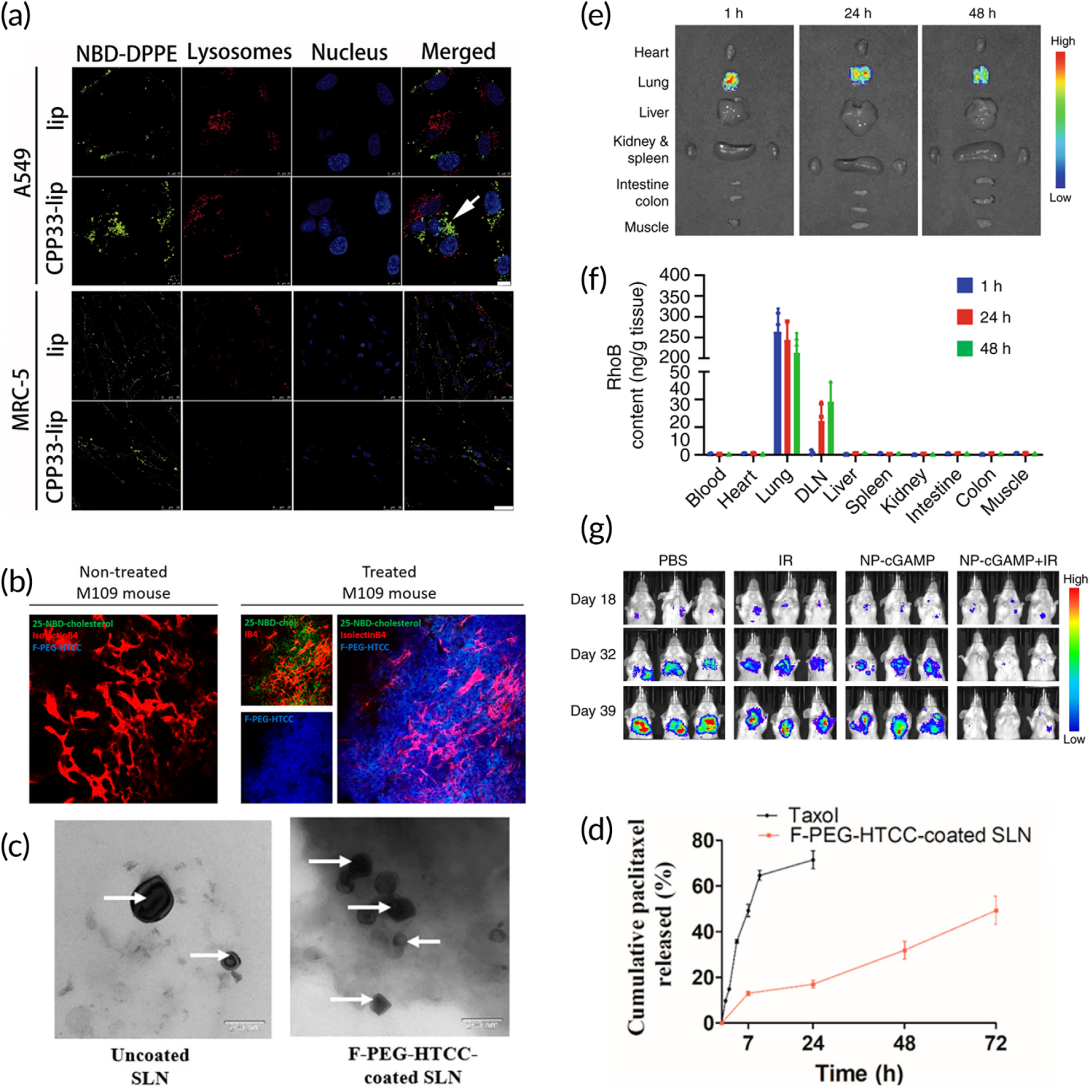
Respiratory track delivery of lipid nanoparticles. (a) Confocal images showing the cellular uptake of NBD‐DPPE‐labeled liposomes (lip) and CPP33‐lip by A549 cells (scale bars, 10 μm) and normal lung fibroblast MRC‐5 cells (scale bars, 50 μm).^97^ (b) Tumor distribution of folate‐PEG‐N‐(2‐hydroxypropyl)‐3‐trimethylammonium chloride chitosan (F‐PEG‐HTCC)‐coated SLN loaded with 25‐NBD‐cholesterol after administration by inhalation. Confocal images of control untreated M109 mouse lungs and coated fluorescent SLN‐treated mouse lungs. Red = vessels labeled with isolectinB4, green = 25‐NBD‐cholesterol labeling the SLNs, blue = Alexa Fluor 405‐grafted‐F‐PEG‐HTCC labeling the coating. (c) TEM images of uncoated and coated SLNs (scale bar is 200 nm, the white arrows are SLN cores). (d) In vitro release profiles of PTX in PBS at 37°C under sink conditions.^98^ (e) Representative ex vivo fluorescence images of major organs dissected from 4T1‐luc lung metastases‐bearing mice at 1, 24, and 48 h post inhalation of DiR‐labeled PS‐coated NPs. (f) High‐performance liquid chromatography measurements of the concentration of PS‐coated NPs labeled with RhoB in various tissues of the 4T1‐luc lung metastasis mice post inhalation. (g) NP‐cGAMP inhalation plus radiation (IR) is efficacious against 4T1 breast cancer lung metastases. NP‐cGAMP was inhaled 24 h after each IR for a total of three inhalations. IVIS images of three representative animals from each treatment group.^95^ Figures are reprinted with publishers' permission.

To ensure that damage to healthy lung cells is minimized, LNPs can also be designed to preferentially target and release drugs in lung cancer cells after inhalation. Targeted liposomes loaded with triptolide, as shown in Figure 5a, were delivered through pulmonary administration to mouse models of non‐small cell lung cancer (NSCLC).^96^ Triptolide has been shown to be cytotoxic to cancer cells and sensitizing to chemotherapy in vitro, however, as it is highly toxic and poorly water soluble, liposomal encapsulation is considered a promising delivery option. Conjugation of anti‐carbonic anhydrase IX (CA IX) antibodies to the surface of liposomes improved tumor targeting, as CA IX is a hypoxia‐inducible enzyme, overexpressed in many cancers with a hypoxic TME,^130^ although antibodies against CA IX alone have not shown significant improvement in lung cancer outcomes.^131^ Liposomal delivery can be measured using an endotracheal tube and was found to sustain drug release in the lungs for up to 96 h and to improve survival in mice. In a subsequent study, the surface of triptolide‐loaded liposomes delivered by pulmonary administration was further modified.^97^ Two ligands were used with the liposomes to treat NSCLC: anti‐CA IX Ab and CPP33, a cell‐penetrating peptide. All liposomes showed low blood levels of triptolide and good lung specificity, with the dual ligand modified liposome showing the best anticancer effect.

Another targeting strategy is to use molecules that strongly bind overexpressed proteins on the surface of lung cancer cells, such as folate receptors.^132^ For example, folate‐grafted chitosan derivatives can be complexed onto paclitaxel‐loaded SLNs to enhance their delivery.^98^ The SLNs were delivered by inhalation to lung tumors in a mouse model of lung carcinoma. The aim of the chitosan derivative was to prolong the lung retention of the SLNs and to be more selective against lung cancer due to the folate groups grafted onto the backbone (Figure 5b–d). Within 1 h, the concentration of paclitaxel‐loaded SLNs in the lungs was 7‐fold higher than the inhaled free paclitaxel, and 32‐fold higher at 6 h. Importantly, the blood plasma concentration of paclitaxel was low for both inhaled free paclitaxel and inhaled SLNs, dropping below the limit of detection immediately for the SLNs and within 1 h for free paclitaxel, suggesting a minimal risk of systemic side effects.

In addition to cytotoxic agents, the local delivery of immunomodulatory drugs also benefits from reduced systemic toxicity, while allowing a more robust immune activation in the lung. Loira‐Pastoriza et al. developed a cationic liposomal oligonucleotide formulation for enhanced antitumor activity in a syngeneic B16F10 mouse model of metastatic lung cancer.^99^ First, the authors tested unmethylated oligodeoxynucleotides containing CpG motifs (CpG), which act as Toll‐like receptor (TLR) 9 agonists. TLRs are important inflammatory signaling receptors that can significantly modulate the anti‐cancer immune response.^133^ Systemic and intratumoral injections of CpG have been previously investigated, and their encapsulation in liposomes has been shown to better activate immune cells.^134^ For this reason, the authors aimed to increase the antitumor efficacy of immune stimulation by using liposomes as vectors. The second drug tested was polyinosinic:polycytidylic acid (poly I:C), which is a double‐stranded RNA that acts as a TLR 3 agonist. The authors found that the liposomes did not encapsulate poly I:C as stably as CpG, but both drugs slowed tumor growth in vivo. CpG liposomes had 28‐fold less tumor cells when administrated locally, but only 3‐fold less tumor cells when the formulation was delivered systemically. Liposomal CpG was better at increasing the cytokine levels in the lung compared with CpG alone, with no significant systemic inflammatory response.

Immunomodulatory nanoparticles can also be targeted to other cell types besides cancer cells, such as immune cells. Liu et al. developed a targeted and inhalable immunomodulatory liposomal system and demonstrated synergistic effects with radiotherapy for long‐term control of lung metastases in mice.^95^ The LNPs were loaded with cyclic guanosine monophosphate‐adenosine monophosphate (cGAMP), which is a stimulator of interferon genes (STING) agonist. The aim was to target antigen‐presenting cells (APCs) in the lungs by coating the liposomes with phosphatidylserine, which allows them to bind to the phosphatidylserine receptors expressed on tissue‐resident APCs (Figure 5e–g). The liposomes were designed to enhance cytosolic release of cGAMP and stimulate STING signaling and type I interferon production in APCs, resulting in a proinflammatory environment. The liposomal STING formulation was combined with fractionated radiation to provide synergistic and systemic effects for anticancer immunity. The system was found to be effectively delivered to the lungs, and repeated tumor challenges demonstrated long‐term survival. The authors also noted that the mass mean aerodynamic diameter of the aerosols was 1.38 μm, which is optimal for deep lung deposition.^135^ Looking more specifically at the synergistic effects with radiotherapy for lung metastases, the authors found that liposomal cGMAP with radiotherapy significantly reduced the number of metastatic foci in the lungs. Only the combination treatment showed complete tumor clearance in some mice, and that APCs and CD8+ T cells were required to induce the anti‐tumor immunity.

### 3.3 Intratumoral and peritumoral delivery

**FIGURE 6:**
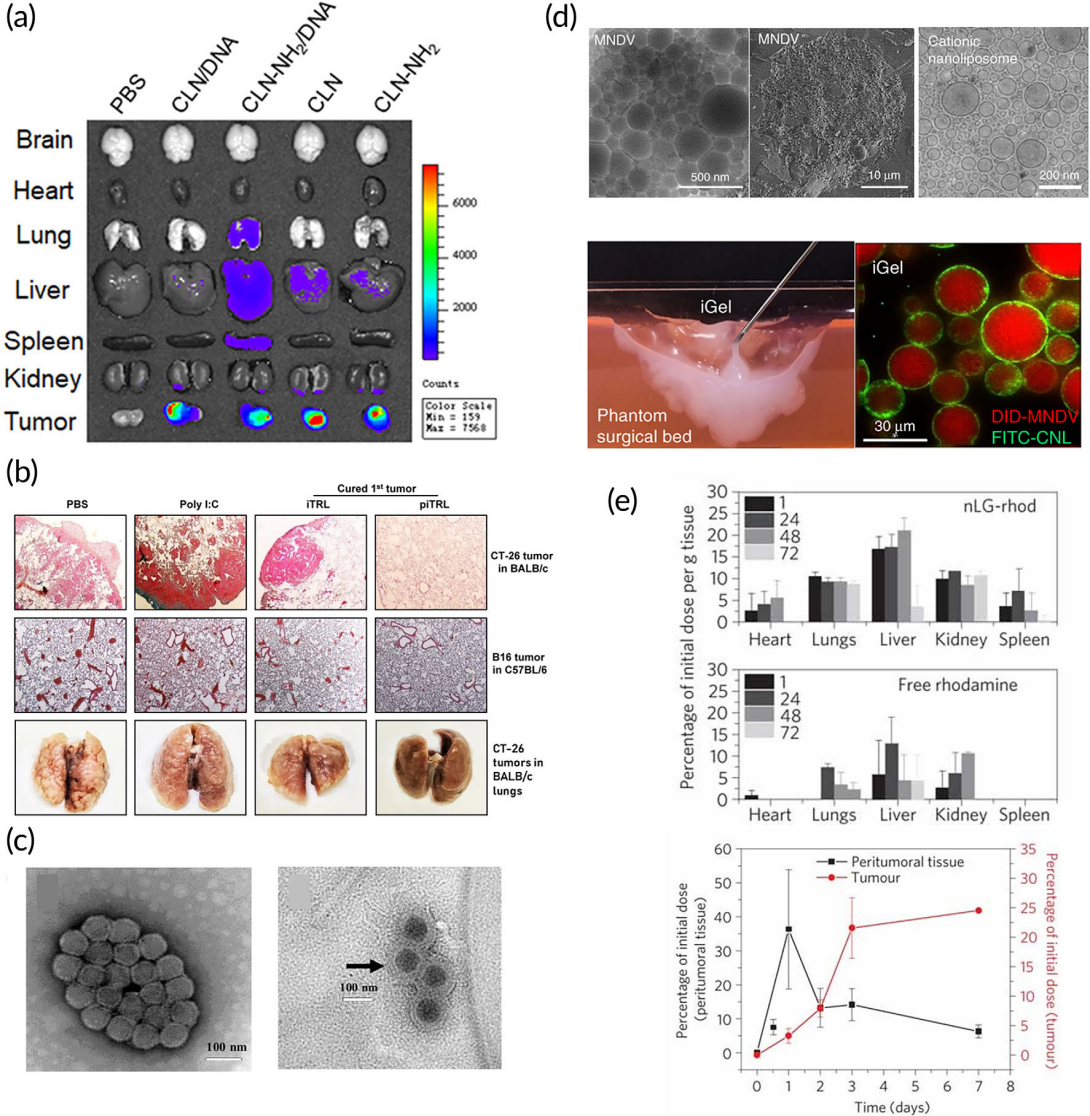
Intratumoral delivery of lipid nanoparticles. (a) Intratumoral injection of CLN/DNA enhancing the DC activation in tumor‐draining lymph nodes in vivo. Fluorescence images of the brain, heart, lung, liver, spleen, kidney, and tumor harvested from CT26 tumor‐bearing mice at 48 h after IT injection of PBS, CLN‐NH2/DiD, CLN/DiD, CLN‐NH2/DiD/DNA, and CLN/DiD/DNA. The dose of plasmid DNA was 369 μg/kg per injection. All nanoparticles were labeled with DiD.^104^ (b) Protective effect of piTRL treatment with laser irradiation against lung metastasis of cancer. On day 28 of the first transplanted tumor challenge, mice treated with iTRL or piTRL and laser irradiation were further intravenously inoculated with CT‐26 and B16 cells, respectively. PBS‐ and poly I:C‐treated mice were also injected with the cancer cells as a control.^102^ (c) Co‐delivery of paclitaxel (PTX) and IL‐12‐expressing adenoviral vector (Ad5). Transmission electron micrograph (TEM) images of naked Ad5 (left) and AL/Ad5/PTX (right). Scale bar = 100 nm.^108^ (d) Syringe‐injected post‐surgical gel depot (iGel) for localized treatment, composed of multi nanodomain vesicles (MNDV) and cationic liposomes.^101^ (e) Liposomal polymeric gel (nLG) with combination delivery of immunotherapy drugs (TGF‐β inhibitor and IL‐2), showing controlled release clearance and biodistribution in healthy animals. Whole body biodistribution: significantly higher amounts of rhodamine were detected in the major organs of nLG‐treated animals (top panel) compared with animals injected with free dye (bottom panel). Data are presented as mean percentage of initial dose given. Time‐dependent accumulation in subcutaneous tumor: cumulative rhodamine tumor penetration (red) after B16 peritumoral injection in B6 mice. Peritumoral tissue was collected to quantify the remaining dose of nLG surrounding the tumor (black).^107^ Figures are reprinted with publishers' permission.

Intra/peritumoral delivery of LNPs by direct injection has the advantage of being highly localized and tissue specific. However, locally delivered nanoparticles are unable to infiltrate distant metastases, which limits this delivery method to tumors that are accessible to injections and that have already been identified. Interestingly, certain intratumoral (IT) treatments have been shown to improve the response of untreated distal tumors due to the priming of a systemic immune response known as the abscopal effect, which is primarily mediated by T cells.^136^ An example of the abscopal effect was shown by Cong et al. who developed an immunomodulatory cationic liposome‐DNA (CLN/DNA) complex.^104^ In a CT26 colon cancer mouse model, this nanoformulation showed significant TME remodeling and immune response through the mobilization of dendritic cells (DC) in the tumor‐draining lymph nodes to activate T cells. Overall, both local and distal tumors were significantly inhibited by the local intratumoral injection of the LNPs (Figure 6a). In a different study, indocyanine green, a photothermal agent, was loaded into liposomes to induce a systemic immune response.^103^ The effect of irradiating indocyanine green‐loaded liposomes was studied in CT26 and MC38 colon cancer models in mice. Irradiation of the liposomal formulation eradicated the primary tumors, but long‐term distant tumor growth was minimally inhibited despite higher CD8+ T cell infiltration. Taking advantage of the compensatory upregulation of certain immune checkpoint biomarkers, a combination of liposomal irradiation and a dual PD‐1 and TIM‐3 checkpoint blockade was then used. The results showed that this combination was able to better inhibit distal tumor growth. Similar findings were reported with a formulation of thermal responsive liposomes (TRL) for the co‐delivery of indocyanine green and poly I:C (piTRL) in mouse models of CT26 colon cancer and B16F10 melanoma using a liposomal formulation (Figure 6b).^102^ The authors found that the treated tumor was eradicated, and that the mice rejected a rechallenge while also preventing metastasis. Additionally, the authors found that poly I:C activated DCs in the tumor‐draining lymph nodes, which allowed for the mounting of a systemic immune response.

Further research on intratumoral injections has shown that immunotherapeutic agents can directly induce apoptosis in cancer cells. For example, liposomal formulations of poly I:C have been shown to have a pro‐apoptotic effect and inhibit tumor growth in BGC‐823 human gastric adenocarcinoma mouse models.^106^ Cao et al. developed a liposomal formulation of co‐encapsulated paclitaxel and an adenovirus vector type 5 encoding IL‐12, as shown in Figure 6c.^108^ The effect on the liposomal co‐delivery was compared with the delivery of each component as a monotherapy in a B16 melanoma mouse model. The liposomal co‐delivery showed the longest survival times and the highest production of IL‐12 and IFN‐γ. In another immunotherapy study, cytokine‐encoding mRNA was delivered intratumorally using LNPs.^111^ It is desirable to limit the expression of various cytokines to the tumor as to avoid systemic immune overactivation. Encapsulation of IL‐12, IL‐27, and GM‐CSF mRNA into LNANs for intratumoral injection showed good survival and tumor inhibition in mice, and no noticeable systemic toxicity was found. Furthermore, the infiltration of immune effector cells was demonstrated to be highly robust using the co‐delivery approach. Localized chemotherapy can also be combined with immunotherapy in LNPs to improve responses.^105^ In this study, the LNPs encapsulated the chemotherapeutic agent doxorubicin with a soluble form of TNF‐related apoptosis‐inducing ligand (TRAIL) anchored onto the nanoparticle surface. TRAIL is a death ligand that is of interest in cancer research but has shown limited clinical results in the past.^137^ By combining TRAIL with doxorubicin, the authors were able to overcome resistance to TRAIL in cancer cell lines. The co‐delivery of TRAIL and doxorubicin in a human HT‐1080 fibrosarcoma mouse model had significantly higher tumor inhibition than TRAIL alone.

A promising delivery approach involves embedding drug‐loaded LNPs in gel depots to modulate release kinetics in a controlled and sustained spatiotemporal manner, which may further reduce systemic toxicity. Song et al. developed a post‐surgical gel depot composed of multi‐nanodomain vesicles (MNDVs) and cationic liposomes that was able to reshape the TME and establish systemic antitumor immunity (Figure 6d).^101^ The MNDVs contain gemcitabine, a chemotherapeutic agent, and R837, a TLR 7 agonist known as imiquimod. The cationic liposomes were loaded with clodronate, another immunomodulatory drug. Electrostatic forces between the positively charged liposomes and the negatively charged MNDVs form a multidomain gel, which was tested in 4T1 breast cancer and TC1 cervical cancer mouse models. First, the tumor was surgically excised, and the gel was implanted directly adjacent to the unresected tumor mimicking cancer‐positive surgical margins. The results showed that the gel conferred systemic antitumor immunity and generated a memory T‐cell response in the process. Further, the combination with a PD‐1 checkpoint blockade therapy showed a synergistic effect in 4T1 mouse models, but not in TC1 models, highlighting the variability in the immune response based on tumor types. Several gel depots also rely on photothermal therapy to control drug release. Won et al. developed an injectable gel depot composed of a chitosan hydrogel containing liposomal doxorubicin.^109^ This system reduces systemic doxorubicin toxicity and significantly inhibits B16F10 melanoma tumor growth. Within the tumor tissue, the system acts as a doxorubicin reservoir, whose release can be controlled by near‐infrared light. Further, the authors found that the combination of the gel with a PLGA nanoparticle vaccine, by which the tumor‐specific antigen TRP2 was delivered to DCs, enhanced the therapeutic efficacy. Another gel containing a combination of gold nanorods, doxorubicin, and maleimide liposomes was introduced.^110^ Upon infrared irradiation, the gold nanorods generated tumor‐associated antigens via immunogenic cell death while the maleimide liposomes would act as antigen capture agents to promote antigen uptake by DCs. Finally, the system was combined with an anti‐PD‐L1 antibody for synergistic immunotherapy resulting in prolonged survival in a B16F10 melanoma mouse model. Tsai et al. evaluated a thermoresponsive gel depot.^100^ Liposomal imiquimod was incorporated into a temperature‐sensitive hydrogel using Pluronic F127. The authors studied the effects of in situ gel injection and compared it to systemic intravenous injections of liposomal imiquimod in a 4T1 breast cancer mouse model. The results showed that mice treated with the gel had the highest survival and good response rates, likely due to the sustained, local release of the drug at the tumor site. Nanoscale gel depots encapsulated within liposomes can be another drug delivery approach, which was used, for example, in the slow and controlled release of a TGF‐β inhibitor and IL‐12 to overcome cancer immune evasion in the TME.^107^ In B16 melanoma mouse models, the gel depot administered by peritumoral injection led to significant drug deposition in the tumor compared with other organs (Figure 6e), and inhibited tumor growth, resulting in prolonged survival of the mice.

## 4 INTRAVENOUS DELIVERY OF LIPID NANOPARTICLES

The intravenous (IV) delivery of nanoparticles is the most commonly used delivery method to date because it allows for the widespread distribution of LNPs throughout the body, and therefore to metastatic tumors. The IV delivery method relies predominantly on the EPR effect to concentrate the LNPs into the TME relative to other healthy tissues. Nevertheless, the major drawback of IV delivery is the sequestration and elimination of most LNPs by the hepatic, immune, and renal systems before they can localize into the tumor. A comprehensive review of the elimination pathways of nanoparticles from the circulation has already been reported by Poon et al.^138^ Various techniques and methodologies for designing lipid nanoparticle formulations and coatings for passive or active delivery have been developed and optimized to improve drug delivery with good results in several cancer types using a large variety of payloads and combinatorial approaches.

### 4.1 Passive delivery of LNPs

LNPs can be formulated using standard phospholipids without significant surface modifications other than PEGylation, which typically improve their pharmacokinetics. These LNPs can be classified as passive delivery systems as they are not designed to bind to specific cells or tissues. Multiple types of passive nanoformulations have been developed to date encapsulating a range of therapeutic cargo summarized in Table 2.

**TABLE 2:** Summary of passive IV delivery formulations for LNP‐based treatments of solid tumors.

Cancer	Cell type	Model	LNP type	Mechanism of action	Treatment			Date	References
					Chemo/targeted therapy	Immunotherapy	Gene therapy		
Breast	4T1.2	Ectopic	Liposome	DNA intercalation, IDO1 inhibition	DOX	NLG919	N/A	2017	[139]
	MDA‐MB‐231		Liposome	Microtubule stabilization	Paclitaxel	N/A	N/A	2018	[140]
	C(3)1Tag	Orthotopic	Liposome	STING‐mediated IFN response + PD‐1 blockade	N/A	cGAMP + αPD‐L1	N/A	2018	[141]
	MCF‐7	Ectopic	LNAN	Apoptosis induction, inhibition of drug resistance proteins	7‐*O*‐geranylquercetin	N/A	microRNA	2019	[142]
	4T1	Orthotopic	Cationic Liposome	DNA alkylation, TLR 7/8 agonism	Oxaliplatin	Resiquimod	N/A	2020	[143]
		Ectopic	Liposome	Immunomodulation	N/A	Urosolic acid	N/A	2020	[144]
	MMTV‐PyMT	Orthotopic	Liposome	PKA inhibition, CTLA‐4 blockade	H89	αCTLA‐4	N/A	2020	[145]
	EMT6, 4T1	Ectopic	Liposome	Topoisomerase inhibition, IDO inhibition	Mitoxantrone	Indoximod	N/A	2020	[146]
	4T1	Orthotopic	Liposome	DNA intercalation, immunomodulation	DOX	Silybin	N/A	2021	[147]
		Orthotopic + Metastatic	Liposome	Histone deacetylase inhibition, PD‐1/PD‐L1 blockade	Chidamide	BMS‐202	N/A	2021	[148]
		Ectopic	Liposome	DNA intercalation, JMJD1A inhibition	DOX	IOX1	N/A	2021	[149]
		Orthotopic	Lipid Nanodisc	STING‐mediated IFN response	N/A	Cyclic dinucleotides	N/A	2022	[150]
	EMT6, 4T1		Liposome	DNA intercalation + angiotensin II receptor antagonism, PD‐1 blockade	DOX + losartan	αPD‐1	N/A	2022	[151]
	4T1		Liposome	Microtubule stabilization, PD‐1/PD‐L1 blockade	Paclitaxel	BMS‐202	N/A	2022	[80]
	4T1, NIH3T3	Ectopic	Liposome	Microtubule stabilization + TGF‐B signaling attenuation	Docetaxel + salvianolic acid	N/A	N/A	2022	[152]
Colon	CT26	Ectopic	Liposome	Induction of DNA strand breaks	Bleomycin	N/A	N/A	2018	[153]
			Liposome	TLR 9 agonism	N/A	CpG	N/A	2018	[154]
			Cationic Liposome	DNA alkylation, TLR 7/8 agonism	Oxaliplatin	Resiquimod	N/A	2020	[143]
		Orthotopic	Lipoplex	Inhibition of VEGF expression	N/A	VEGF siRNA + Cu^2+^	N/A	2020	[155]
		Ectopic	Liposome	Topoisomerase inhibition, IDO inhibition	Mitoxantrone	Indoximod	N/A	2020	[146]
			Liposome	DNA alkylation, IDO1 inhibition	Oxaliplatin	NLG919	N/A	2020	[156]
	WiDr		Liposome	TLR 7/8 agonism + EGFR blockade	N/A	Resiquimod + αEGFR	N/A	2021	[157]
	CT26		Liposome	DNA intercalation, JMJD1A inhibition	DOX	IOX1	N/A	2021	[149]
	MC38		Lipid Nanodisc	STING‐mediated IFN response	N/A	Cyclic dinucleotides	N/A	2022	[150]
	CT26		Liposome	DNA alkylation +	Oxaliplatin + Metformin	αPD‐1	N/A	2022	[158]
			Liposome	Immunomodulation + PD‐1 blockade	N/A	All‐trans retinoic acid + αPD‐1	N/A	2022	[159]
Fibrosarcoma	WEHI‐164	Ectopic	Liposome	DNA intercalation	DOX	N/A	N/A	2022	[160]
Liver	SK‐HEP‐1	Ectopic	Liposome	Microtubule stabilization	Paclitaxel	N/A	N/A	2018	[140]
Lung	TC‐1	Ectopic	Lipid Nanodisc	STING‐mediated IFN response	N/A	Cyclic dinucleotides	N/A	2022	[150]
	A549		Liposome	Immunomodulation + PD‐1 blockade	N/A	All‐trans retinoic acid + αPD‐1	N/A	2022	[159]
Melanoma	B16	Metastatic	Liposome	STING‐mediated IFN response	N/A	Cyclic di‐GMP	N/A	2015	[161]
		Ectopic	Liposome	DNA alkylation, TLR 9 agonism	Cisplatin	CpG	N/A	2016	[162]
			Liposome	STING‐mediated IFN response, PD‐L1 blockade	N/A	cGAMP + αPD‐L1	N/A	2018	[141]
			Liposome	TLR 9 agonism	N/A	CpG	N/A	2018	[154]
			Liposome	DNA intercalation, immunomodulation	DOX	Tumor lysate	N/A	2019	[163]
			LNAN	Tyrosine kinase signaling antagonism, inhibition of PD‐L1 expression	Imatinib	N/A	siPD‐L1	2020	[164]
			Liposome	DNA intercalation, CTLA‐4 blockade	DOX	αCTLA‐4	N/A	2020	[165]
			Cationic Liposome	DNA alkylation, TLR 7/8 agonism	Oxaliplatin	Resiquimod	N/A	2020	[143]
			Liposome	DNA intercalation + immunogenic cell death	Anthracyclines+ Shikonin	N/A	N/A	2021	[166]
		Metastatic	Liposome	STING‐mediated IFN response	N/A	Cyclic di‐GMP	N/A	2021	[167]
		Ectopic	Liposome/LNAN	DNA intercalation, inhibition of PD‐L1 expression	DOX	N/A	siPD‐L1	2021	[168]
			Liposome	DNA intercalation + iron chelation	DOX + Deferasirox	N/A	N/A	2022	[169]
	PDX, B16F10		LNAN	Fas‐mediated cell death	N/A	Fas plasmid	N/A	2022	[170]
Ovarian	SKOV3	Ectopic	Liposome	Microtubule stabilization	Paclitaxel	N/A	N/A	2018	[140]
Prostate	RM‐1	Ectopic	Liposome	DNA intercalation + immunogenic cell death	Anthracyclines+ Shikonin	N/A	N/A	2021	[166]
Renal	RENCA	Ectopic	Liposome	Topoisomerase inhibition, IDO inhibition	Mitoxantrone	Indoximod	N/A	2020	[146]

#### 4.1.1 Chemotherapy

The first LNP formulation approved by the FDA was liposomal doxorubicin (Doxil®) in 1995. Doxorubicin is a chemotherapeutic agent used against many cancers, and it works by intercalating DNA and inhibiting cell replication.^171^ Unfortunately, like most chemotherapeutic agents, doxorubicin has significant dose‐limiting side effects, such as cardiotoxicity,^172^ but its utility in oncology drove the motivation for developing a liposomal formulation to improve the drug's safety profile. Initial iterations of liposomal doxorubicin were met with significant pharmacokinetic challenges, until the circulation time was optimized using PEGylation, effectively increasing its half‐life in plasma. Doxil® has been shown to reduce cardiotoxicity while having equivalent therapeutic efficacy when compared with doxorubicin alone in multiple cancers, which forms the foundation of its clinical approval and use.^173^ Today, formulations of liposomal doxorubicin may vary due to the development and approval of various generic lipid carriers. A study by Smith et al. evaluated the clinical activity of two liposomal doxorubicin formulations, the original liposomal formulation (Doxil®) and a generic version (Lipodox®).^174^ The retrospective study was conducted in response to a shortage of available Doxil® and tested the toxicity and response rate in patients with recurrent ovarian cancer. The authors found that Lipodox® had a similar toxicity profile to Doxil® but was less effective, with response rates of 4.3% and 18%, respectively, warranting a prospective study for a head‐to‐head comparison that could better explain the observed differences.

Irinotecan (SN‐38) is another important liposome‐encapsulated chemotherapeutic drug that works by inhibiting topoisomerase I leading to cell death in rapidly dividing cells.^175^ Phase I and II clinical trials examined the nanoliposomal irinotecan formulation PEP02 in various advanced refractory solid tumors.^176^, ^177^ The safety and pharmacokinetic profile were established, along with promising preliminary results on clinical efficacy. A subsequent phase III trial led to the approval of liposomal irinotecan in combination with 5‐FU and folinic acid for the treatment of pancreatic adenocarcinoma.^178^ Cisplatin, another chemotherapeutic, is commonly used as the first‐line treatment of stage III and IV non‐small‐cell lung cancer (NSCLC) along with other agents and works by crosslinking DNA.^179^ Liposomal formulations have been studied in preclinical and clinical trials to reduce the systemic toxicity of cisplatin. In a phase III clinical trial, liposomal cisplatin in combination with paclitaxel (described below) was shown to be as effective as the treatment with free cisplatin and paclitaxel.^180^ In addition, the systemic toxicity of the liposomal formulation was significantly reduced. Liposomal cisplatin showed negligible nephrotoxicity, nausea, peripheral neuropathy, and asthenia. A 2018 meta‐analysis of clinical trials that directly compared liposomal cisplatin with conventional cisplatin supported its reduced toxicity, and even found improved efficacy, based on the progressive disease rate metric, for both NSCLC and squamous cell carcinoma (SCC) of the head and neck.^181^

Paclitaxel, a chemotherapeutic agent within the taxane family, is also commonly used in a wide variety of cancers. Taxanes work primarily by binding to tubulin and stabilizing microtubules in cancer cells thereby preventing their normal function.^182^ Due to its hydrophobicity, the commercial formulation (Taxol®) solubilizes paclitaxel using Cremophor® EL to be injectable as an aqueous solution. However, the inclusion of Cremophor® EL has been associated with a variety of adverse side effects, allergic reactions, and changes in the toxicity profile of the active chemotherapeutic agent.^183^ A liposomal paclitaxel formulation was developed and compared with Taxol® by Huang et al. to overcome some of these limitations.^140^ The study used xenograft tumor models of breast (MDA‐MB‐231), ovarian (SK0V3), and liver (SK‐HEP‐1) cancers in mice. They found that the liposomal paclitaxel formulation had equivalent efficacy to Taxol®, with reduced side effects such as hematopoietic toxicity, acute hypersensitivity reactions, and cardiac arrhythmias, suggesting that the paclitaxel‐liposomal formulation may have greater therapeutic value. Furthermore, clinical trials comparing various lipid‐based formulations of paclitaxel or docetaxel support the hypothesis that liposomal formulations in general maintain clinical efficacy while reducing toxicity.^184^, ^185^ Unfortunately, not all cancers respond to chemotherapy alone, requiring the development of other therapeutic avenues that are complementary to the use of chemotherapy.

#### 4.1.2 Immunotherapy

A major goal of immunotherapies is to induce an anticancer immune response and to remodel the TME with the goal of improving the immunogenicity of cancer cells. Resiquimod (R848), a TLR 7/8 agonist, has been shown to significantly alter the TME in a mouse model of pancreatic cancer leading to improved outcomes.^186^ Similarly, liposomes encapsulating resiquimod have been shown to remodel the TME by the re‐education of TAMs in the tumor tissue of WiDr colon cancer models in mice, especially when combined with an anti‐EGFR antibody.^157^ Na et al. used a liposomal formulation of a macrophage modulator to enhance tumor regression and eliminate pro‐tumoral TAM functions.^145^ H89 was used as a protein kinase A (PKA) inhibitor, as PKA overactivation in TAMs contributes to various aspects of cancer progression.^145^ When combined with an anti‐CTLA‐4 antibody for immune checkpoint inhibition, the immunotherapeutic effect was significantly enhanced, and CD8+ T‐cells expanded by ~60‐fold.

**FIGURE 7:**
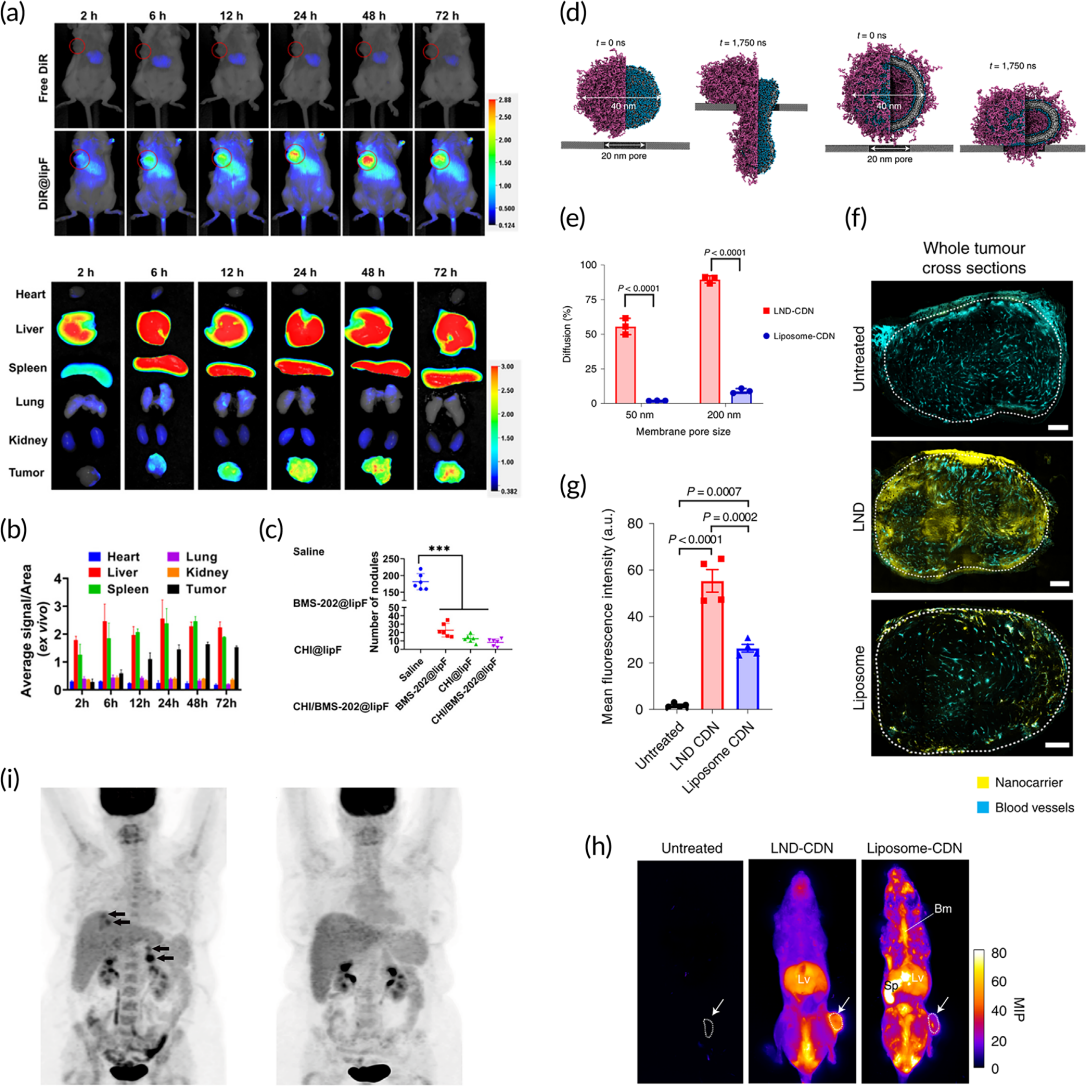
Passive intravenous delivery of lipid nanoparticles. (a) Biodistribution and anti‐metastatic effects of liposomal immunotherapy, using co‐delivered chidamide (CHI) and BMD‐202. Representative fluorescence images of mice at different time points; red circle shows the site of the tumor. (b) Quantification of average signal intensity of various mouse organs showing the time‐dependent accumulation of LNPs into the tumor tissue. (c) CHI/BMS‐202@lipF‐mediated anti‐metastasis effect in the 4T1 lung metastasis model showing the number of lung nodules.^148^ (d) Delivery of STING agonist cyclic dinucleotides (CDN) using lipid nanodiscs (LND). Coarse‐grained simulation snapshots of an LND (left) and a liposome (right), both with a diameter of 40 nm, before (*t* = 0 ns) and after (*t* = 1750 ns) being pulled through a 20 nm pore. (e) LND‐CDN demonstrates superior passive diffusion compared with liposome‐CDN in vitro. Shown is the percentage of particles detected in the receiver chamber after 24 h after diffusion through a membrane with pore sizes of 50 and 200 nm. (f) Representative whole tumor (MC38) cross sections and enlarged views of tumor vessels from mice treated with Cy5‐labeled LND or liposome (yellow). Scale bars = 50 μm. (g) Mean fluorescence intensities averaged from four tumor regions of interest per mouse. (h) Representative maximum intensity projections of whole mice with tumors identified with a white arrow.^150^ (i) Phase I clinical trial of LNP‐encapsulated tumor suppressor gene, showing DC‐TUSC2 metabolic tumor response in a metastatic lung cancer patient. The first image is the pretreatment PET scan. Second image is the post‐treatment PET scan performed 20 days following the fourth dose of DC‐TUSC2.^188^ Figures are reprinted with publishers' permission.

In another study, Tu et al. aimed to develop a liposomal formulation to increase TME immunogenicity and facilitate a response to PD‐L1 immune checkpoint inhibition.^148^ The formulation contained chidamide, an epigenetic modulator that can induce immunogenic cell death, and BMS‐202, a small molecule PD‐L1 inhibitor. Using a 4T1 breast cancer mouse model, the authors found a time‐dependent accumulation of the formulation in the TME resulting in a synergistic immune response that was able to inhibit tumor growth in primary and spontaneously metastatic tumors as shown in Figure 7a–c.^148^ Zheng et al. investigated how a liposomal formulation of all‐trans‐retinoic acid (ATRA) can remodel the immunosuppressive TME by inducing MDSC differentiation and maturation.^159^ They developed a sustained release formulation of actively loaded ATRA. Using a CT26 colon cancer mouse model, the authors found that liposomal ATRA alone, and in combination with immune checkpoint PD‐1 inhibitors, significantly reduced tumor growth. Similarly, crystalline ursolic acid in a liposomal formulation also demonstrated remodeling of the immunosuppressive environment but had limited antitumor efficacy in a 4T1 breast cancer mouse model.^144^

Many immunotherapy approaches use a STING agonist, such as liposomal CpG for use in colon cancer and melanoma,^154^, ^162^ liposomal cyclic diguanylate (di‐GMP) in melanoma,^161^, ^167^, ^187^ and liposomal cGAMP for breast cancer.^141^ Dane et al. developed a novel approach of using lipid nanodiscs to carry cyclic dinucleotides (Figure 7d).^150^ The lipid nanodiscs were able to better penetrate tumors due to their more elastic and flexible architecture (Figure 7e–h) and performed significantly better than liposomes for long‐term tumor remission, and T‐cell priming for antitumor immunity.

#### 4.1.3 Chemoimmunotherapy

Typically, immune checkpoint blockade therapies such as anti‐PD‐1 and anti‐CTLA‐4 are not as effective in poorly immunogenic (cold) tumors compared with immunogenic (hot) tumors.^189^ A recent study found that the combination of liposomal doxorubicin, losartan, and anti‐PD‐1 therapy in 4T1 breast cancer mouse models showed improved immunogenicity and enhanced antitumor immune activity with overall reduced tumor volume.^151^ In this case, doxorubicin causes immunogenic cell death, thereby facilitating further immune responses, while losartan reduces the density of the stroma, allowing the liposomal doxorubicin to better penetrate the tissue. Similarly, the combination of docetaxel and salvianolic acid B encapsulated in liposomal carriers improved the therapeutic outcomes in a mouse model of breast cancer.^152^ Salvianolic acid B was shown to remodel the TME by interfering with CAFs, thereby enhancing the chemotherapeutic effects of PEGylated liposomal docetaxel.

Alendronate has been shown to remodel the TME toward higher levels of immunogenicity by depleting TAMs.^190^ A study by Islam et al. evaluated the effects of a PEGylated liposomal formulation of doxorubicin and alendronate in a mouse fibrosarcoma model.^160^ The results indicated a greater modulatory effect on the immunogenicity of the TME with co‐encapsulated drugs compared with liposomal doxorubicin alone, particularly with respect to the myeloid cell populations such as TAMs, MDSC, T_reg_, and NK cells. Other formulations containing silybin^147^ or 5‐carboxy‐8‐hydroxyquinoline (IOX1), a histone demethylase inhibitor^149^ in combination with doxorubicin or checkpoint inhibitors show TME remodeling capabilities. One study examined the effect of the administration sequence on combination therapies.^165^ The authors found that a liposomal formulation of anti‐CTLA‐4 increased survival and reduced tumor size in B16 melanoma mouse models compared with free anti‐CTLA‐4. However, the combination with Doxil® demonstrated synergistic effects when anti‐CTLA‐4, whether in liposomal formulation or free, was administered before Doxil®, underlining that the sequence of therapy administration is an important parameter to consider for effective therapeutic design in combination therapy.

Song et al. designed a liposomal formulation to deliver oxaliplatin and metformin to the tumor.^158^ The authors used an oxaliplatin prodrug conjugated to 1,2‐distearoyl‐sn‐glycero‐3‐phosphatidylethanolamine (DSPE) as the lipid component, whereas metformin was encapsulated in the liposomal core. The role of metformin was to reduce the hypoxic changes present in the TME, thus increasing the immunogenic cell death induced by oxaliplatin. When combined with anti‐PD‐1 immune checkpoint blockade therapy in a CT26 colon cancer mouse model, liposomal oxaliplatin and metformin exhibited a marked anti‐tumor immune response. A similar liposomal formulation of oxaliplatin in combination with NLG919, an IDO1 inhibitor was also developed.^156^ IDO1 is an enzyme that can drive immunosuppression in the TME via the kynurenine pathway to suppress effector T cell function.^191^ The combination demonstrated effective chemoimmunotherapy in a murine colorectal cancer model. Mei et al. found that a PEGylated liposomal formulation of mitoxantrone, a chemotherapeutic drug, and indoximod, an IDO1 inhibitor, showed improved survival rates and a synergistic effect on the immune response for several murine cancer models, including CT26 colon cancer, EMT6 and 4T1 breast tumors, and RENCA kidney tumors.^146^ To incorporate indoximod into the liposome, the drug was conjugated to cholesterol and integrated into the lipid bilayer.

#### 4.1.4 Gene therapy

Gene therapy can block the translation of various proteins and enzymes that are essential for cancer development, survival, and growth. For example, Li et al. combined a siRNA against PD‐L1 with the receptor tyrosine kinase inhibitor imantinib, which is already used to treat various cancers.^164^ Imantinib was incorporated into the lipid shell of the nanoparticle, while the negatively charged siRNA was complexed with positively charged PEI in the core of the LNP since the siRNA/PEI complex confers stability and controlled delivery of the siRNA. The co‐delivery to murine B16 melanoma models showed significant tumor growth inhibition compared with single or combined non‐liposomal components underlining the importance of drug co‐delivery at predetermined ratios. A complementary study also looked at a B16 melanoma model in mice using siRNA‐based PD‐1 silencing.^168^ The authors combined the effects of the PD‐1 siRNA with Doxil®, which significantly improved survival over the LNP siRNA alone. Other relevant anticancer siRNA targets include survivin, IL‐10, or transmembrane serine protease 4 (TMPRSS4), which may play a role in cancer cell survival, metastasis, migration, and adhesion.^142^, ^197^

Plasmid DNA (pDNA) transfection can be used to generate in situ therapeutic protein production that could last longer than mRNA‐based production, which may require multiple injections. Charoensit et al. developed liposomal complexes containing ATRA and pDNA encoding IL‐12, a proinflammatory cytokine associated with anti‐neoplastic activity, against metastatic lung cancer of CT26 colon cancer cells in mice.^198^ The liposomal complex reduced the number of tumor cells and nodules in the lungs and significantly increased the survival time of the mice. Multiple clinical trials are currently evaluating various forms of LNP‐based formulations for the treatment of solid tumors (summarized in Table 3). Unfortunately, human trials are inherently more variable and complicated to perform compared with pre‐clinical animal models. While most trials can achieve some of their secondary endpoints, such as good biodistribution or immune stimulation, many fail to reach significance with respect to their primary endpoints, such as overall survival or progression‐free survival, compared with the standard of care; especially when it comes to Phase II and III trials. This leads to the preemptive termination of trials that evaluate formulations, which are neither safe nor significantly better than the standard of care, or that are not economically viable. For example, a phase I clinical trial of a lipid nanoparticle‐encapsulated tumor suppressor gene‐containing plasmid (TUSC2/FUS1) was carried out in patients with recurrent and metastatic lung cancer.^188^ The formulation was well‐tolerated and safe. Plasmid delivery was observed in both primary and metastatic tumors, as shown in Figure 7i, with some gene expression, pathway alterations, and consequently antitumor effects being noted by the authors. However, more robust trials would be needed to confirm the efficacy of this formulation and validate these findings.

**TABLE 3:** Summary list of clinical trials involving LNPs for the treatment of solid tumors.

Name	LNP type	Drug	Phase	Initiated	Status	NCT/ref
MBP‐426	Liposome	Oxaliplatin; transferrin	Phase I/II	2009	Unknown	NCT00964080
EphA2‐targeting DOPC‐encapsulated siRNA	LNP	EphA2 siRNA	Phase I/II	2012	Recruiting	NCT01591356
2B3‐101	Liposome	Doxorubicin	Phase I/II	2011	Completed (2014)	NCT01386580, NCT01818713
ThermoDox	Liposome	Doxorubicin; MR‐HIFU induced hyperthermia	Phase I	2015	Recruiting	NCT02536183
		Doxorubicin; superficial hyperthermia; radiation	Phase II	2016	Withdrawn	NCT02850419
		Doxorubicin; radiofrequency ablation	Phase III	2008	Completed (2017)	NCT00617981
		Doxorubicin; MR‐HIFU induced hyperthermia	Phase I	2018	Unknown	NCT03749850^192^
		Doxorubicin; radiofrequency ablation	Phase III	2014	Completed (2018)	NCT02112656
		Doxorubicin; MR‐HIFU induced hyperthermia	Phase II	2021	Recruiting	NCT04791228
		Doxorubicin; focused ultrasound	Phase I	2021	Withdrawn	NCT04852367
		Doxorubicin; focused ultrasound	Phase I	2014	Completed (2019)	NCT02181075^193^, ^194^
Tecemotide (L‐BLP25) vaccine	Liposome	Tecemotide	Phase III	2006	Completed (2015)	NCT00409188^195^
MM‐302	Liposome antibody conjugate	Doxorubicin; HER2‐targeted antibody	Phase I	2016	Withdrawn	NCT02735798
		Doxorubicin; HER2‐targeted antibody; trastuzumab	Phase II/III	2014	Terminated (2016)	NCT02213744^196^
		Doxorubicin; HER2‐targeted antibody	Phase I	2011	Unknown	NCT01304797
ONT 10	Liposome	Glycopeptide MUC1 targeted antigen; PET lipid A adjuvant	Phase Ib	2014	Completed (2016)	NCT02270372
MM‐310	Liposome	Docetaxel prodrug; EphA2 receptor targeting	Phase I	2017	Unknown	NCT03076372
mRNA‐2752	LNP	OX40L, IL‐23, and IL‐36γ mRNA	Phase I	2018	Recruiting	NCT03739931
TKM‐080301	LNP	PLK1 siRNA	Phase I/II	2011	Completed (2012)	NCT01437007
DepoCyt	Liposome	Ara‐C	Phase I/II	2019	Terminated	NCT01044966
SOR007	Topical LNP	Paclitaxel	Phase I/II	2017	Completed (2021)	NCT03101358^117^
mRNA‐2416	LNP	OX40L mRNA	Phase I/II	2017	Terminated	NCT03323398
LiPlaCis	Liposome	Cisplatin	Phase I/II	2013	Completed (2021)	NCT01861496
SGT‐53	Liposome	p53 cDNA; transferrin receptor‐targeted	Phase I	2021	Withdrawn	NCT05093387
PIPAC‐NAL‐IRI^a^	Liposome	Irinotecan	Phase I	2022	Recruiting	NCT05277766

^a^ Pressurized intraperitoneal aerosol chemotherapy.

### 4.2 Targeted delivery

**FIGURE 8:**
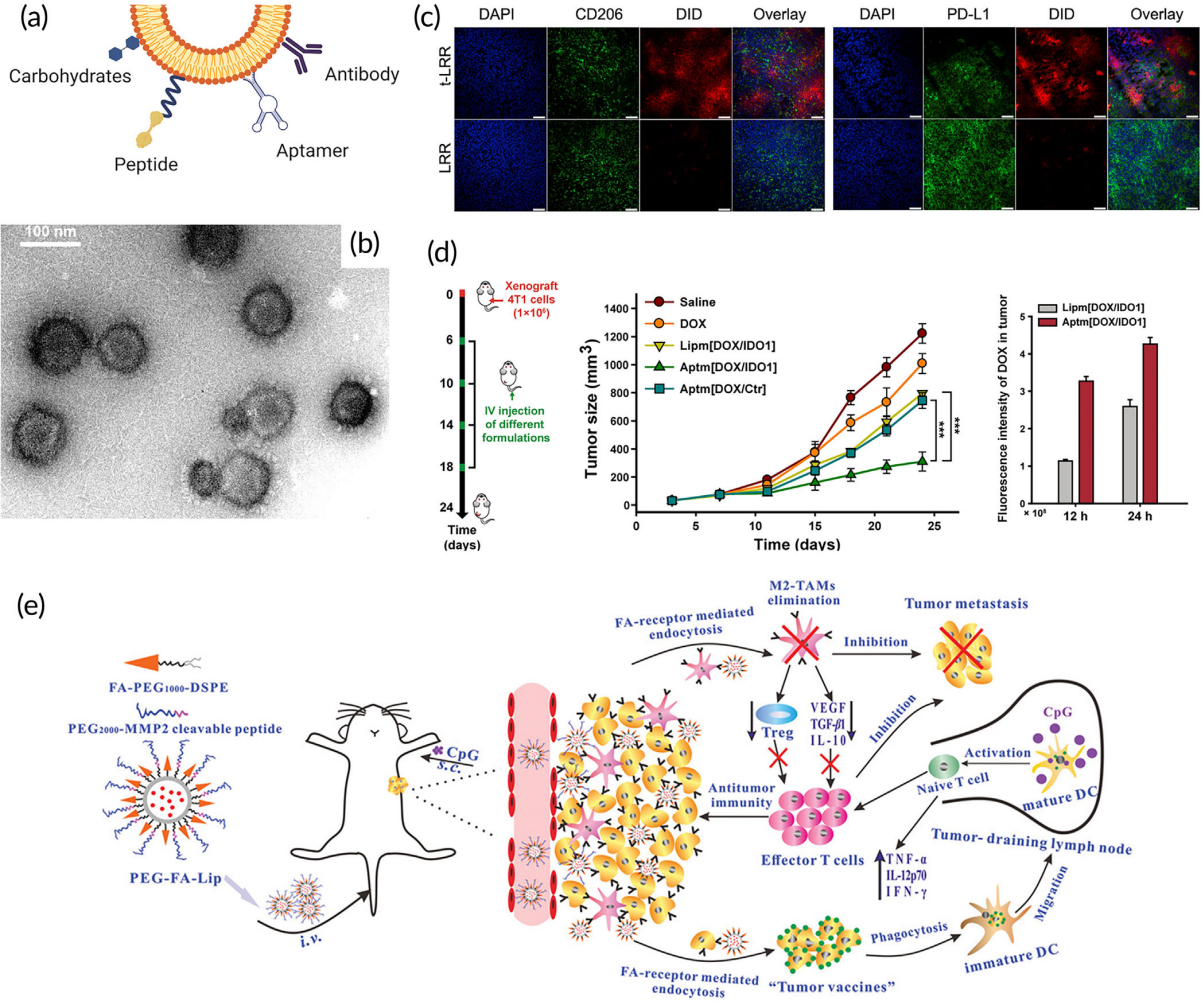
Intravenous delivery of targeted lipid nanoparticles. (a) Various types of targeting moieties that can be grafted onto lipid nanoparticles. (b) TEM images showing doxorubicin‐loaded LNPs functionalized with the targeting ligands cyclic RGDfK and peptide‐22.^222^ (c) Ex vivo immunofluorescence images showing the co‐localization of targeted and non‐targeted LRRs with CD206 and PD‐L1 markers.^236^ (d) Efficacy and intratumoral accumulation of fluorescently labeled aptamer‐based anti‐CD44 and anti‐PD‐L1 targeted and non‐targeted liposomes containing doxorubicin and siRNA against IDO1.^246^ (e) Mechanism of action of the PEG‐FA‐Lip construct for chemoimmunotherapy.^200^ Figures are reprinted with publishers' permission.

One of the main advantages of LNP carriers is that they can be easily adapted for active targeting to tissues or cells of interest by conjugating their surface with targeting moieties (Figure 8a). This strategy is used to improve the accumulation of LNPs at their target sites to avoid off‐target toxicity and improve the therapeutic efficacy of the formulation, and while it can also be used for local delivery, it holds tremendous promise to improve the biodistribution profile of LNPs after IV administration. Various chemical ligands can be grafted onto the surface of LNPs (summarized in Table 4) to enable the binding of the nanoparticles to the surface of target cells, resulting in their internalization and intracellular delivery via endocytic pathways.^199^ TME‐associated cells, including cancer cells, typically exhibit aberrant molecular signaling, endowing them with properties that can be exploited for active targeting, such as the overexpression of certain surface receptors.

**TABLE 4:** Examples of targeting strategies used for LNPs in the treatment of solid cancers.

Targeting method	Targeting moiety	Target	Cell type	References
Small Molecule	Folate	Folate receptor	Multiple cancers, TAMs	[200, 201]
	Mannose	Mannose receptor	TAM, DC, colorectal cancer	[202, 203, 204, 205, 206, 207]
	Sialic acid	Siglec‐1 selectin	TAM breast cancer	[208, 209, 210, 211]
Peptide	LFC131	CXCR4	Liver cancer	[212]
	SP5‐2	VEGFR1	NSCLC	[213]
	H1299.3	N/A	NSCLC	[214]
	TMT	Aminopeptidase	Metastatic cancer	[215]
	T7 A7R	TfR VEGFR2	Glioma	[216]
	WH	Clec9a	DCs	[217]
	rlipoE7m	TLR 2	APCs	[218]
	A20FMDV2	Integrin α_v_β_6_	Cancer cells and TME	[219]
	RGD motifs	Integrin α_v_β_3_	Cancer cells and TME	[220, 221, 222, 223, 224]
Antibody	mAb	CD44	Ovarian cancer	[225]
	2C5 mAb	Nucleosome	Breast cancer	[226]
	scFv G8 Hyb3	MAGE‐A1 via HLA‐A1	Melanoma	[227]
	mAb	HER2	Breast cancer	[228]
	scFv	EphA2	Cancer cells and TME	[229]
	scFv	TfR	Cancer cells and TME	[230, 231, 232]
	Fab, mAb, sdAb	EGFR	Cancer cells and TME	[197, 233, 234, 235]
	mAb, sdAb	PD‐L1	Cancer cells and TME	[236, 237, 238, 239, 240]
	mAb	NKp46	NK cells	[241]
	mAb	DICR	DCs	[242]
	Fab	CD3	T cells	[243]
Aptamer	DNA AS1411	Nucleolin	Melanoma	[244]
	RNA Apt2	CD44	Cancer cells and TME	[245, 246]
	DNA Aptm	PD‐L1	Cancer cells and TME	[246]
	RNA	IL‐4R	Cancer cells and TME	[247]
	DNA ENG‐Apt	Endoglin	Cancer cells & TME	[248]

#### 4.2.1 Peptide ligands

Small peptide ligands can be developed to target specific surface molecules on cancer or immune cells, usually through phage display technology, which allows a large number and variety of peptides to be screened against specific molecules.^249^ For example, phage display was used to identify several peptides that preferentially target NSCLC cells compared with normal bronchial epithelium, which can be used to develop targeted drug‐loaded liposomes with impressive results.^213^, ^214^ Other peptides have been developed and formulated with LNPs that can specifically bind to metastatic cells,^215^ vascular endothelial growth factor receptor 2,^216^ or other relevant cancer cell receptors and immune cell receptors for immunomodulation.^212^, ^217^, ^218^ An important peptide sequence that has been extensively investigated for its ability to bind to integrins is the arginine–glycine–aspartic acid (RGD) motif. Peptides containing this motif have been used to develop targeted LNPs to various cells in the TME, which may be of particular interest for bone metastases and brain tumors.^220^, ^221^, ^224^, ^250^, ^251^ For example, Chen et al. developed a dually functionalized doxorubicin‐loaded liposome containing cyclic RGDfK and peptide‐22, which binds the low‐density lipoprotein receptor (LDLR) as shown in Figure 8b. This construct was able to cross the blood–brain barrier and accumulate in a brain glioma mouse model, resulting in the improved survival of the mice.^222^

#### 4.2.2 Antibody‐based ligands

Antibodies and nanobodies, which are single‐domain antibodies, are of great interest for ligand targeting, as they are stable in circulation and can bind strongly to specific epitopes. Amongst their many applications in biology and medicine, antibodies have also been used to target nanoparticles to specific cell receptors expressed on cancer cells, such as the transferrin receptor (TfR),^230^, ^231^, ^232^ EGFR,^197^, ^233^, ^234^, ^235^ HER2,^228^ EphA2,^229^ CD44,^225^, ^252^ nucleosome‐restricted,^226^ MHC‐restricted antigens,^227^ for chemoimmunotherapy. Interestingly, IgG antibodies formulated with LNPs seem to accumulate in tumors to a greater extent than free antibodies.^253^ Similar to cancer cells, immune cells can also be targeted, for example, NK cells via NKp46,^241^ DCs via DICR,^242^ or adoptive T cells via Thy1.1^254^, ^255^ for immunomodulation. Notably, the immune checkpoint molecule PD‐L1 is frequently expressed on cancer cells and has been studied as a potential drug delivery ligand for theranostic and combination therapy.^237^, ^238^, ^239^, ^240^ An interesting design was the development of a PD‐L1‐targeted liposome encapsulating rapamycin and regorafenib (LRR) for the combined inhibition of mTOR signaling and angiogenesis resulting in the metabolic reprogramming of the TME.^236^ The authors were able to improve the nanoparticle delivery to the tumors compared with non‐targeted controls, as shown by ex vivo immunofluorescence in Figure 8c, while also reducing tumor growth and improving survival in colorectal cancer mouse models. However, there are some limitations concerning antibody‐tagged LNPs, as they do not seem to significantly improve the nanoparticle's biodistribution profile.^256^

#### 4.2.3 Aptamer ligands

Aptamers are typically single‐stranded oligonucleotides, often <30 kDa in size, that contain unique molecular architectures that allow them to bind specific molecules with very high affinity.^257^ Aptamers are isolated from a large pool of random oligonucleotides through a process known as the systematic evolution of ligands through exponential enrichment (SELEX), making their development and production more affordable than monoclonal antibodies.^258^ They can be conjugated on the surface of LNPs in more complex supramolecular structures^259^ to target the molecular cargo to specific cells or proteins, such as endoglin or the IL‐4 receptor, but extensive validation of binding affinities is required, since LNP‐bound aptamers may not retain the same structure as aptamers free in solution.^244^, ^245^, ^248^, ^260^, ^261^ Nonetheless, aptamers have shown promise as targeting agents, demonstrated by Kim et al. using liposomes loaded with doxorubicin and siRNA against IDO1.^246^ The authors grafted two aptamers on the surface of liposomes targeting both CD44 and PD‐L1, effectively creating actively targeted liposomes carrying a triple combination of targeted, chemo and immunotherapies. The LNP construct was able to accumulate in tumors to a high extent and significantly slow 4T1 breast cancer tumor growth in mice as shown in Figure 8d.

#### 4.2.4 Small molecule ligands

Folate receptors can be significantly overexpressed on multiple types of epithelial cancer cells compared with normal cells.^262^ Efforts have been made to conjugate folate to the surface of LNPs to improve their targetability and uptake. For example, folate receptor‐targeted liposomes were developed for the co‐delivery of doxorubicin along with siRNA against Bmi1, a protein involved in cancer cell proliferation and development.^201^ The authors showed that the targeted liposomes were deposited in the tumors to a greater extent, resulting in a significant reduction in tumor volume in an SCC xenograft mouse model (KB cells). In another study, folate‐conjugated liposomes were used for the delivery of doxorubicin, which was adjuvanted with CpG peritumorally, to target both cancer cells and macrophages for TME immunomodulation (Figure 8e).^200^ The study showed that targeted liposomes accumulated predominately in tumor tissues and resulted in a significant tumor growth reduction of both primary and distant tumors in a breast cancer mouse model (4T1). Two other important receptors that can be expressed on both cancer and immune cells are the mannose and sialic acid receptors. LNPs have been synthesized with both mannose^202^, ^203^, ^204^, ^205^, ^207^, ^263^ and sialic acid^209^, ^210^, ^211^ derivatives to target the TME for cytostatic or immunotherapy. In one instance, mannosylated liposomes were loaded with a human papillomavirus type 16 E7 peptide along with CpG, which induced a strong immune response in the TME leading to TC‐1 tumor shrinkage in mice.^206^ Li et al. developed a liposomal drug delivery system containing sialyated cholesterol and loaded it with doxorubicin and metformin for chemoimmunotherapy.^208^ The assessment of the formulation in mouse models of breast cancer (4T1) and metastatic melanoma (B16F10) showed direct accumulation of the LNPs into the tumors. When combined with anti‐PD‐1 antibodies, the combination significantly reduced tumor growth and improved survival in mice.

## 5 TRIGGERABLE RELEASE SYSTEMS

Another approach to control drug release and delivery from lipid nanoparticles is to design triggerable release systems as part of the LNP construct. Upon exposure to a specific stimulus in the TME, coated or conjugated molecules as part of the LNPs are modified or cleaved, facilitating drug delivery into the TME and to cancer cells. A variety of release mechanisms have been developed to date, including formulations that respond to pH, hypoxia, or external energy.

### 5.1 pH

**FIGURE 9:**
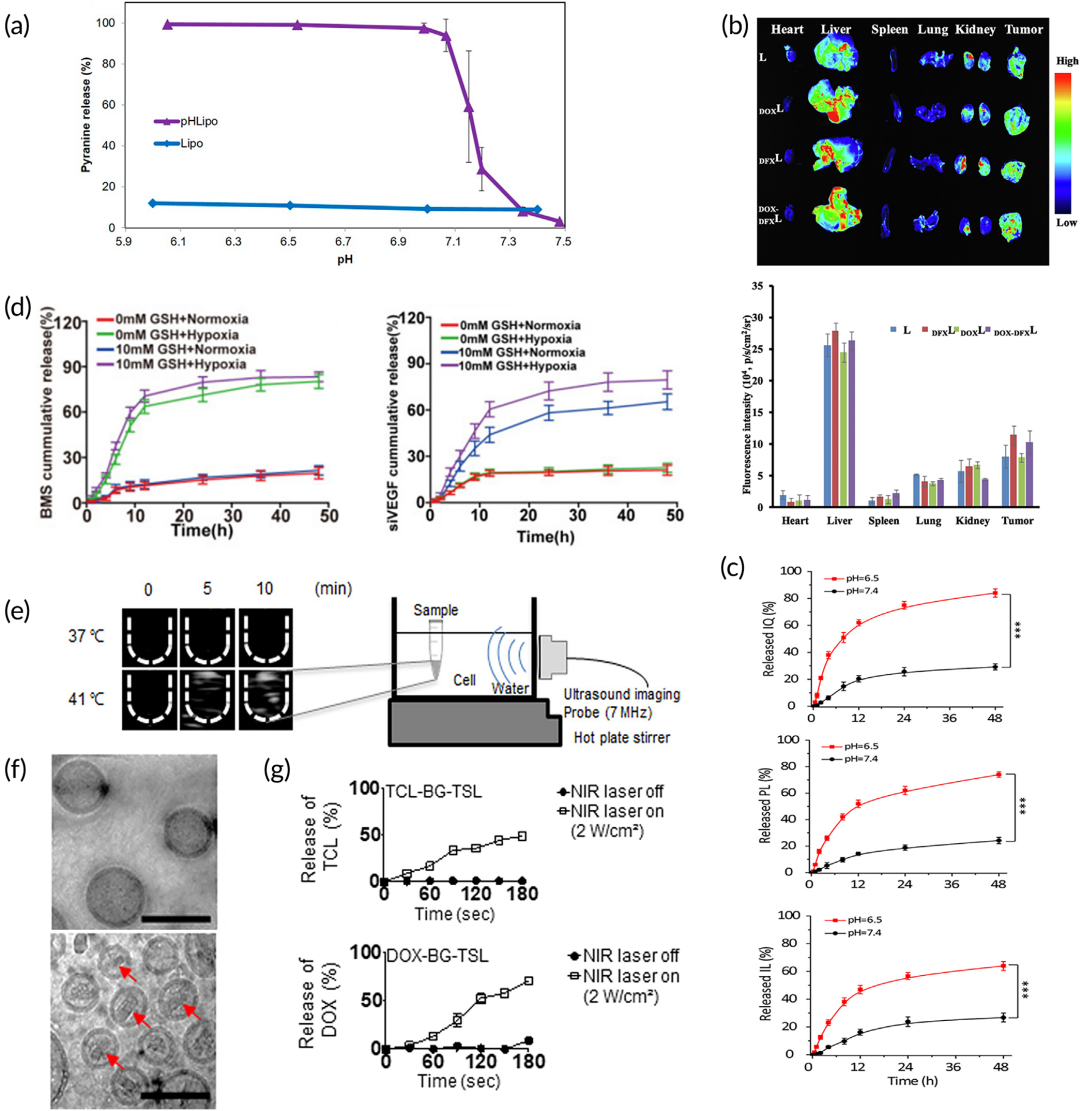
Triggered release delivery of lipid nanoparticles. (a) Delivery of bleomycin with pH‐sensitive liposomes quantified via pyranine release from liposomes modified with or without CHexPG‐PE at 37°C across a range of pH values.^153^ (b) Fluorescence images and their quantification of excised organs and tumors at 24 h post‐injection of L‐R, DOXL‐R, DFXL‐R, and DOX‐DFXL‐R.^169^ (c) The release profile of imiquimod (IQ), anti‐PD‐L1 (PL), and IL2 (IL) from pH responsive T_reg_‐tagged liposomes.^265^ (d) Drug release profiles from HLBBRT in different conditions.^155^ (e) Liposomal carrier system for tumor cell lysate with photothermal irradiation‐triggered CO_2_ bubble generation. (f) Morphologies of BG‐TSLs and DOX‐BG‐TSLs monitored by cryoTEM. The arrow denotes DOX encapsulation. Scale bar = 100 nm. (g) Release of DOX or TCL from TSLs or BG‐TSLs with or without NIR irradiation (2 W/cm^2^).^163^ Figures are reprinted with publishers' permission.

As the TME is generally more acidic than normal tissue, pH‐sensitive coatings and complexes have been developed to control payload release from lipid nanoparticles.^153^, ^166^, ^169^, ^264^, ^265^, ^266^ Yuba et al. used pH‐sensitive liposomes (Figure 9a) to enhance the uptake of bleomycin in tumor cells and inhibit CT26 colon tumors in mice.^153^ To confer pH sensitivity, the authors modified the liposomes with the pH‐reactive 2‐carboxycyclohexane‐1‐carboxylated polyglycidol distearoyl phosphatidylethanolamine (CHexPG‐PE). The modified liposomes were shown to be taken up by tumor cells at a 2.5‐fold higher rate, however, the construct seemed to induce toxicity in the spleen, liver, and lungs when a high concentration of bleomycin was delivered. The toxicity to the spleen and liver was partially alleviated by increasing the molecular weight of the PEG chains on the surface of the liposomes. Another chemoimmunotherapy liposome formulation was developed similarly to the ones described earlier, but with an additional pH‐sensitive coating to release a dual drug payload.^169^ Deferasirox (DFXL), an inhibitor of the PI3K/Akt pathway, was co‐encapsulated with doxorubicin and the pH sensitivity was conferred by the inclusion of cholesteryl‐hemisuccinate (CHEMS) on the surface of liposomes, where the carboxylate groups of the CHEMS are protonated in an acidic environment leading to bilayer instability and drug release. In a B16F10 melanoma mouse model, deferasirox and doxorubicin‐loaded LNPs accumulated in tumors (Figure 9b), improved the anti‐tumor immune response, and significantly inhibited tumor growth. Ou et al. developed immunotherapeutic liposome formulations co‐loading imiquimod, IL2, and anti‐PD‐L1 antibodies that were tagged onto T_reg_ cells via an anti‐CD25 antibody Fab to be trafficked into the TME.^265^ Since the liposomes were fabricated using 1,2‐dioleoyl‐sn‐glycero‐3‐phosphoethanolamine (DOPE), they exhibited pH sensitivity leading to a significant release of the cargo at an acidic pH, as shown in Figure 9c. The authors concluded that by exploiting this trafficking and release mechanism, they were able to achieve significant tumor response rates in B16 melanoma mouse models and noted a significant activation of DCs and infiltration of CD8+ T cells, mediating a strong antitumor response.

### 5.2 Hypoxia and other methods

Lower oxygen concentration is another characteristic of the TME that can be exploited for controlled drug delivery. Chen et al. developed a hypoxia‐triggerable liposome “bioreactor” (or HLBBRT) that demonstrated synergistic antitumor immunity in a CT26 mouse colorectal cancer model.^155^ A hypoxia‐sensitive nanoparticle shell was constructed by the self‐assembly of commercially available soybean phospholipids and a hypoxia‐sensitive azobenzene derivative. For the bioreactor component, the release of Cu^2+^ catalyzed chemodynamic therapy in situ to induce tumor damage, thus allowing for differential release kinetics of the encapsulated therapeutics as shown in Figure 9d. Additionally, siRNA against VEGF was complexed within the Cu^2+^ ion‐based nanocomplex to silence angiogenesis. The hypoxia‐triggered bioreactor was found to modulate the proangiogenic‐mediated immunotolerance, alter the tumor endothelium, and synergized with the antitumor immune response generated by the chemodynamic therapy.

In another study, a chemo‐immunoliposome incorporating a mechanism to allow PEG shedding was developed to induce tumor repolarization.^143^ The authors used a combination of liposomal oxaliplatin and resiquimod, while PEGyltion was performed to increase the circulation time and enhance the intratumoral accumulation via the EPR effect. This system allowed for protease‐induced shedding of the PEG coating in the TME, revealing a cationic liposome surface to improve intratumoral trafficking into immunosuppressive MDSCs. The cleavable PEG was anchored to the liposomal membrane by cholesterol and contained four negatively charged glutamic acid residues that can be cleaved by proteases, such as matrix metalloproteinases, expressed in solid tumors.^267^ In a CT26 colon cancer model in mice, the PEG‐shedding liposomal oxaliplatin formulation performed better than a standard formulation, with higher bioavailability and depletion of immunosuppressive TAMs and MDSCs in the TME. In combination with R848, there was improved antitumor immunity and overall therapeutic efficacy. Finally, Won et al. developed a thermosensitive liposomal (TSL) formulation encapsulating doxorubicin and tumor cell lysates (TCL) derived‐antigens that can be released at elevated temperatures after exposure to NIR irradiation (Figure 9e–g) by generating CO_2_ bubbles, resulting in a burst release of the encapsulated cargo. This strategy resulted in significant antitumor immunity in mouse models of melanoma.

## 6 LIMITATIONS AND FUTURE OPPORTUNITIES

While the field has realized significant success in the development of safer chemotherapy formulations, challenges remain to improve the overall efficacy of LNP formulations. One of the most important limitations, especially for targeted delivery of LNPs, is the inconsistent delivery and therapeutic efficacy in human trials.^268^, ^269^, ^270^, ^271^ The striking discrepancy between the success of nanotherapeutics in preclinical studies compared with clinical trials underlines significant drawbacks in the use of current quantitative methods, including animal tumors models, to study LNP targeting in complex tissue environments. Ectopic allogeneic or xenogeneic mouse models are not always representative of human tumors, especially when it comes to assessing the biodistribution of nanoparticles in subcutaneously established tumors due to the lack of characterization of the intratumoral vascular morphology at different time points, as well as significant structural and functional differences with respect to orthotopic/ectopic mouse models and human cancers.^272^, ^273^ The use of more representative models that mimic the cellular heterogeneity and TME features of human cancers should be considered especially for metastatic tumors. For instance, the recent development of prime editing technology may allow the development of cancer mouse models that are more representative of human cancers by recapitulating de facto driver mutations present in patients.^274^ A thorough comparison between current animal model TME and human TME vasculature should be undertaken to identify relevant features or biomarkers that may be more predictive of clinical biodistribution and response rates, since nanoparticle transport into the TME appears to be highly dependent on the cancer type and anatomical location of the tumor, which can vary widely across species.^275^ Alternatively, tumor‐on‐a‐chip models are currently being developed to help bridge the gap between preclinical and clinical discrepancies by helping with the development of more predictable nanoparticle targeting and transport approaches.^276^, ^277^ Recently, an interesting study using theranostic doxorubicin‐loaded and HER2‐targeted liposomes showed that drug deposition in metastatic breast cancer tumors varied significantly even within the same patient, depending on the tumor type and location.^278^ The molecular basis for the differential accumulation of nanoparticles in some tumors, but not others, remains to be elucidated. The authors also highlighted a potential relationship between treatment outcomes and drug deposition, where tumors with more drug accumulation were associated with a better response rate, illustrating the potential of using theranostics to quantify drug accumulation in the tumors as a surrogate marker for predicting efficacy in a clinical setting. An interesting note is that most injected nanoparticles in this study accumulated in the liver and spleen of the patients, replicating some of the observations noted in mouse models.

**FIGURE 10:**
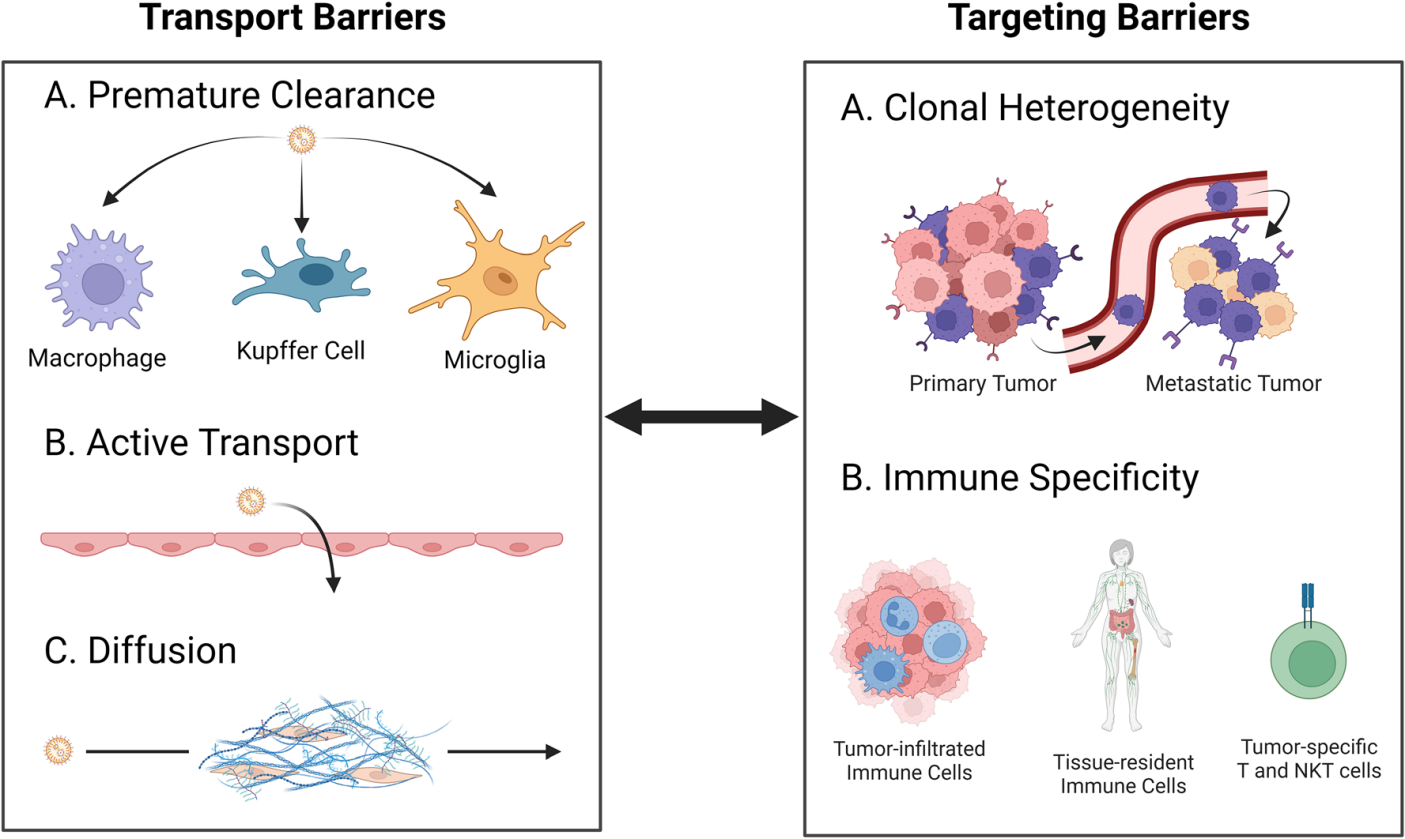
Schematic diagram of transport and targeting constraints on the development of lipid nanoparticles for the treatment of solid tumors. Future nanoparticle designs need to consolidate multiple types of barriers that limit the effectiveness of the encapsulated therapeutics as, for example, adding targeting ligands on the surface of nanoparticles can result in the recognition of nanoparticles by macrophages and lead to their accelerated clearance in the liver or spleen. Created with BioRender.com.

The excessive accumulation of nanoparticles in the liver and spleen remains a major limitation of LNPs, even with active targeting. Studies have estimated that between 30% and 99% of IV‐administered nanoparticles are sequestered in the liver or spleen, with as little as 0.0014% of the injected dose reaching cancer cells for some nanoparticle formulations.^279^, ^280^ There is an urgent need to develop formulations with an adequate circulation half‐life that can rapidly accumulate in primary and metastatic tumors, and subsequently target various immune and cancer cells that reside within those tumors. The design of smart nanotherapeutics will require a balanced approach, where on one hand, nanocarriers must have adequate circulation time and good diffusion across physical tumor barriers, while also targeting very specific TME cell populations, which may require active transport across tight endothelial barriers (Figure 10).^281^ This is a major challenge for the nanomedicine community, as tumors are highly complex, heterogeneous, and dynamic tissues that contain a variety of immune, stromal, and cancer cells, which may differ based on their intrinsic anatomical location, and whether there is the presence of systemic immunity that is already established against cancer cell antigens.^282^ However, various strategies can be employed to design more complex and responsive nanocarriers that can behave differently when in circulation compared with when present in the TME. When nanoparticles are injected intravenously, they acquire a protein corona that endows them with a dynamic biological identity mediated by the Vroman effect.^283^ This new identity can govern their biological fate by altering their cellular uptake and targeting capabilities.^284^, ^285^, ^286^ Recent efforts have attempted to modify or adapt the proteins adsorbed onto LNPs by modifying the chemistry of surface phospholipids to alter their cell and tissue tropism with applications in cancer therapy.^287^, ^288^, ^289^ Another way to adapt synthetic LNPs to biological environments is to modify their surface using biomimetic materials that can be delivered to tumors, such as TME‐derived cell membranes or vesicles.^290^, ^291^ An interesting “walking dead” approach was the conjugation of dead breast cancer cells with anti‐PD‐1 and doxorubicin‐loaded liposomes to increase their delivery to tumors showing good accumulation in 4T1 lung metastases.^292^ Alternatively, nanoparticles can be directly conjugated in/ex vivo onto cells like red blood cells,^293^ macrophages,^294^, ^295^ or lymphocytes^296^, ^297^ to enhance their delivery and reduce premature clearance, a concept known as cell hitchhiking. For example, Siegler et al. attached paclitaxel‐loaded LNPs onto CAR‐NK cells to improve their therapeutic efficacy in a xenograft cancer mouse model showing encouraging results, especially since CAR cell therapies tend to be less effective for treating solid tumors.^298^ The full‐fledged deployment of smart LNP delivery systems has been somewhat hampered due to their complexity and the limited flexibility in changing the chemical composition of the lipid portion of the liposomal formulations. Fortunately, hybrid lipid–polymer nanoparticles may provide a great opportunity to design more clinically relevant nanoparticles through taking advantage of the flexibility of chemical modification of the polymers and the versatility of lipids for delivery of more complex drugs. These various approaches have yet to be fully evaluated in clinical trials, but they hold great promise for improving nanobased cancer therapeutics in the future.

## 7 CONCLUSION

Lipid nanoparticles (LNPs) have attracted considerable resources for designing nanoformulations and understanding their applicability in the field of cancer therapy, which led to important advances in the development of safer chemotherapy treatments and advanced drug delivery applications. While some knowledge gaps and translational limitations remain for their full deployment in the clinic as a first‐line therapeutic modality in solid tumors, the advances to date have helped to establish a solid grounding for the development of next‐generation LNPs, whether for cancer vaccination, immunoengineering, or personalized and precision medicine. The most important challenge is to build nano vectors that can stably encapsulate at least one API and direct the cargo to its intended tissue target. Further advances in targeting chemistry need to consider the complex transport pathways that face nanoparticles in vivo, as well as develop targeting strategies, tissue‐on‐a‐chip, or animal models that take most, if not all, of these parameters into account. Improved circulation time and cell tropism may open the door for their use even beyond oncology as highly specific delivery vehicles for a myriad of applications in genetic engineering and regenerative medicine.

## AUTHOR CONTRIBUTIONS

**Radu A. Paun:** Conceptualization (lead); data curation (lead); methodology (lead); visualization (lead); writing – original draft (lead). **Sarah Jurchuk:** Conceptualization (supporting); data curation (supporting); methodology (supporting); visualization (supporting); writing – original draft (supporting); writing – review and editing (supporting). **Maryam Tabrizian:** Conceptualization (lead); funding acquisition (lead); methodology (supporting); project administration (lead); supervision (lead); validation (supporting); writing – review and editing (supporting).

## CONFLICT OF INTEREST STATEMENT

The authors declare no conflict of interest.

### PEER REVIEW

The peer review history for this article is available at https://www.webofscience.com/api/gateway/wos/peer‐review/10.1002/btm2.10601.

## Data Availability

Data sharing is not applicable to this article as no datasets were generated or analyzedduring the current study.
